# Carnivorous dinosaur lineages adopt different skull performances at gigantic size

**DOI:** 10.1016/j.cub.2025.06.051

**Published:** 2025-08-04

**Authors:** Andre J. Rowe, Emily J. Rayfield

**Affiliations:** 1 School of Earth Sciences, University of Bristol, Bristol BS8 1RJ, UK

## Abstract

Theropoda is one of the most extensively studied dinosaur clades, including iconic carnivores such as *Tyrannosaurus rex* and *Spinosaurus aegyptiacus*. The clade includes the largest terrestrial bipeds ever described, including three lineages that independently achieved giant size: Megalosauroidea, Allosauroidea, and Tyrannosauroidea. Here, we investigate how increasing size influenced feeding performance by quantifying feeding-induced mechanical performance across numerous large theropods using 3D finite element analysis. Unexpectedly, we discovered a divergence in functional strategy among the three lineages that led to gigantic top predators: in non-tyrannosauroid theropods, skull stress generally did not increase with size, in contrast to tyrannosauroids, which experienced greater stress due to increased muscle volume and bite forces. When skulls were scaled to equivalent size, smaller theropods, particularly basal taxa, experienced higher stresses. Despite similar scaling constraints, theropods adopted two distinct functional and likely ecological strategies: increased size with reduced stress or increased skull size, muscle volume, and bite force at the cost of higher stress. Giant tyrannosaurids uniquely maximized bite force despite elevated cranial stress, a strategy perhaps driven by the demands of subduing increasingly large and mobile prey in the Late Cretaceous. Alternatively—or additionally—this shift may reflect ecological displacement by coexisting predators such as smaller theropods and giant crocodyliforms. Whatever the cause, tyrannosaurids pursued a high-risk, high-reward feeding strategy unlike any seen in their Early Cretaceous counterparts, underscoring a profound shift in mega-carnivore evolution near the end of the Mesozoic.

## Introduction

Theropod dinosaurs are among the most iconic fossil organisms, known for their repeated evolution of large body size and for producing the largest bipedal carnivores in Earth’s history.^1–4^ Unambiguous theropods appeared in the Late Triassic (~228 Ma),^5^ and by the Early Jurassic, genera such as *Dilophosaurus* were the largest terrestrial predators.^6^ Multiple theropod clades independently evolved giant forms by the Late Cretaceous, including the ~5,600-kg allosauroid *Acrocanthosaurus*^7,8^ and the ~8,000-kg megalosauroid *Spinosaurus*.^9^

Body size influences many aspects of organismal biology, affecting predation, energetics, and extinction risk.^10–13^ Although Cope’s rule—size increase over time—is not universal across vertebrates, it appears to apply to several theropod lineages.^3,14^ Nonetheless, small-bodied theropods persisted throughout the Mesozoic, reflecting persistent ecological differentiation and functional diversity among carnivorous dinosaurs.^15,16^ Such coexistence likely involved partitioning of prey size, hunting strategies, or habitat use, reducing direct competition with their larger counterparts. At least three major theropod clades independently evolved large size: megalosauroids, allosauroids, and tyrannosauroids.^17,18^ These shifts occurred across time and geography and involved significant changes in skull morphology. For example, basal tyrannosauroids were lightly built (~125 kg), whereas *Tyrannosaurus* reached ~9,500 kg with deep, massively muscled skulls.^19–22^

Although previous studies have examined how size affects locomotion,^23,24^ feeding biomechanics at large size remain less understood. Bite force scales with body mass in extant diapsids,^25–28^ but many prior theropod studies have relied on 2D shape approximations.^29–33^ Modern 3D technologies— computed tomography (CT), surface scanning, photogrammetry—now allow detailed analyses of skull mechanics using finite element analysis (FEA) across a broad taxonomic sample.^34–42^ Yet, fundamental questions remain: how does skull performance scale with body size? Do large theropods reduce stress via geometric scaling or do muscle forces increase proportionally, leading to higher stress loads? Does cranial morphology evolve to offset these forces?

**Figure 1:**
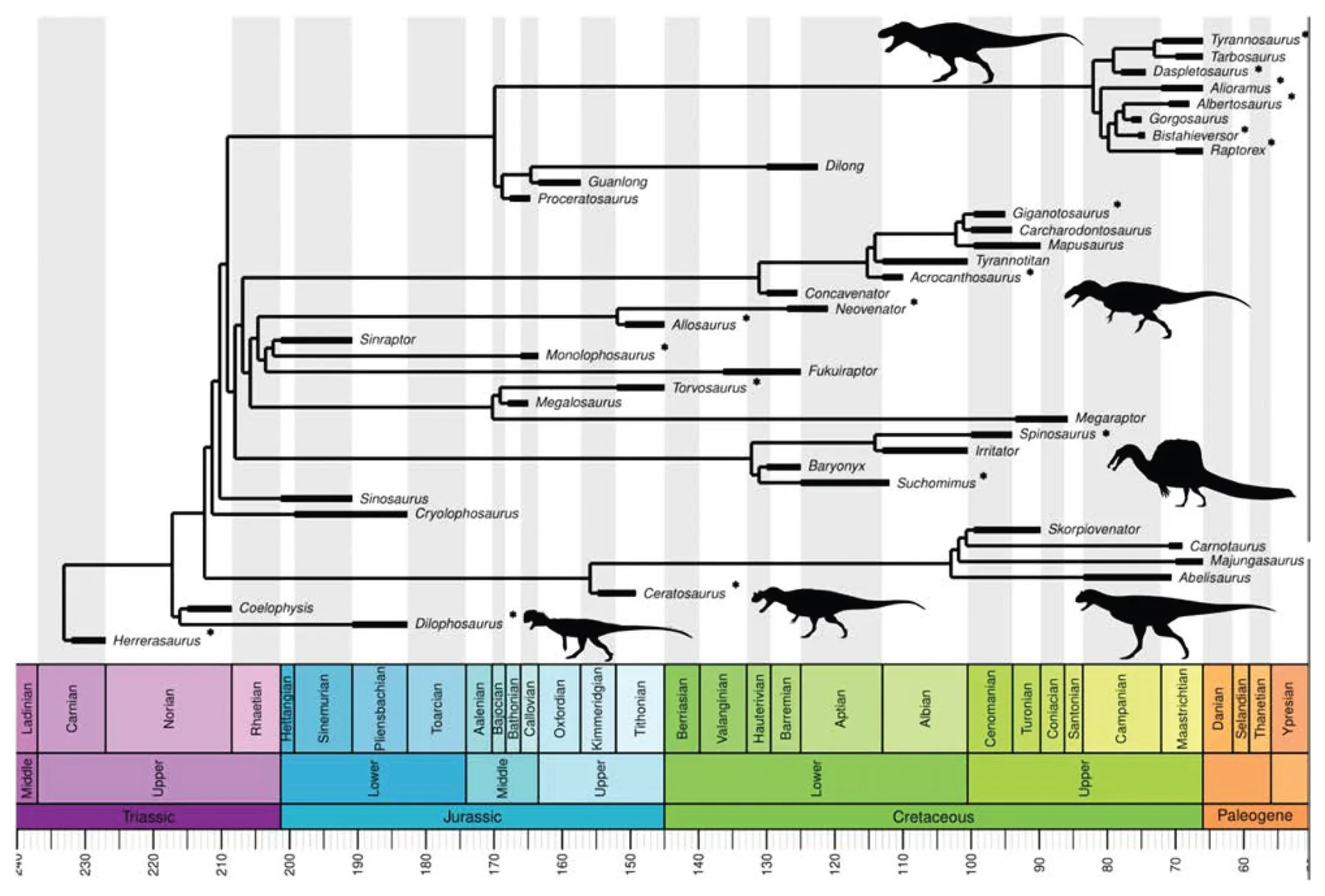
Temporally calibrated theropod dinosaur phylogeny Phylogeny based on several recent studies (Brusatte and Carr,^45^ Hendrickx et al.,^46^ and Tanaka et al.^47^). Asterisks indicate taxa included in this study. Theropod silhouettes from PhyloPic.

Here, we used FEA to test how body size relates to cranial biomechanics across 18 theropods spanning key clades that independently evolved large size (Megalosauridae, Allosauroidea, and Tyrannosauroidea), along with smaller basal taxa such as *Herrerasaurus* and *Dilophosaurus* for comparison (Figure 1). We excluded likely herbivorous theropods like therizinosaurs, which possessed specialized tooth and muscle arrangements adapted for herbivory.^43,44^

We test three key hypotheses using FEA-based 3D mechanical models: (1) stress reduction with size—larger taxa should experience lower stress under geometric scaling predictions; (2) elevated stress in tyrannosauroids—if bite forces scale disproportionately in tyrannosaurids, they should show higher cranial stress relative to similarly sized taxa; and (3) higher stress in smaller taxa when size corrected—when skulls are scaled to the same size, smaller taxa should show higher stress due to less robust skull geometry.

## Results

### Crania at actual size

**Figure 2:**
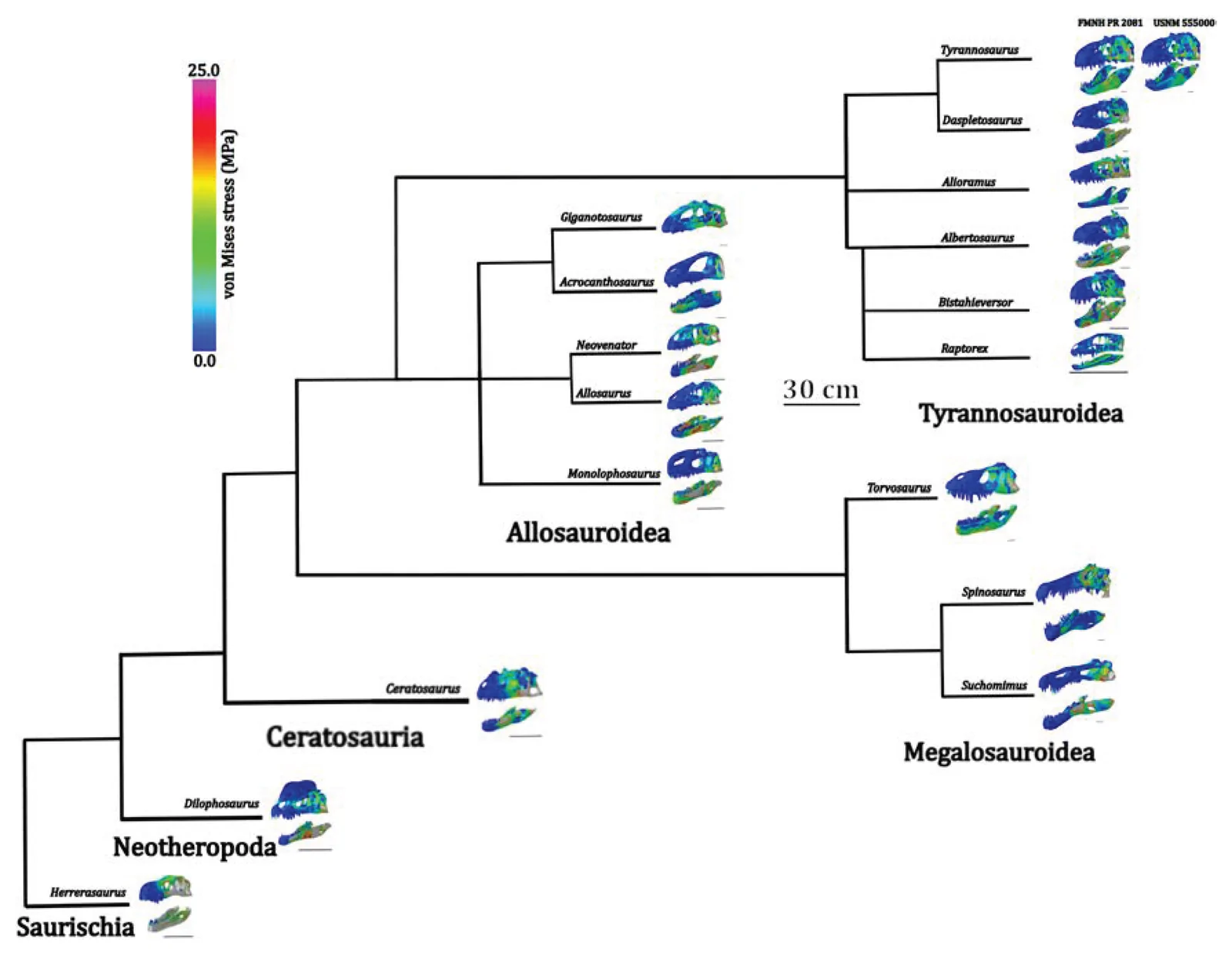
Finite element stress maps for all theropod skulls modeled at actual size after simulated adductor contraction and biting at the two front teeth Areas of high stress are indicated by hotter colors, and low stress is indicated by cooler colors. Gray regions indicate regions with the highest probabilities of structural failure. Note the very high stresses occurring in the basal ambiguous theropod *Herrerasaurus*. For larger stress maps of actual-size skulls, see Figure S1.

Von Mises stress patterns were broadly consistent across taxa (Figure 2), with *Herrerasaurus* displaying the highest cranial stresses, particularly at the rear of the cranium. Large tetanurans such as *Acrocanthosaurus* and *Suchomimus* experienced relatively low cranial stress overall. High-stress areas were concentrated around the quadrate and jugal bones, especially in large tyrannosaurids and allosauroids (*Allosaurus* and *Neovenator*).

**Figure 3:**
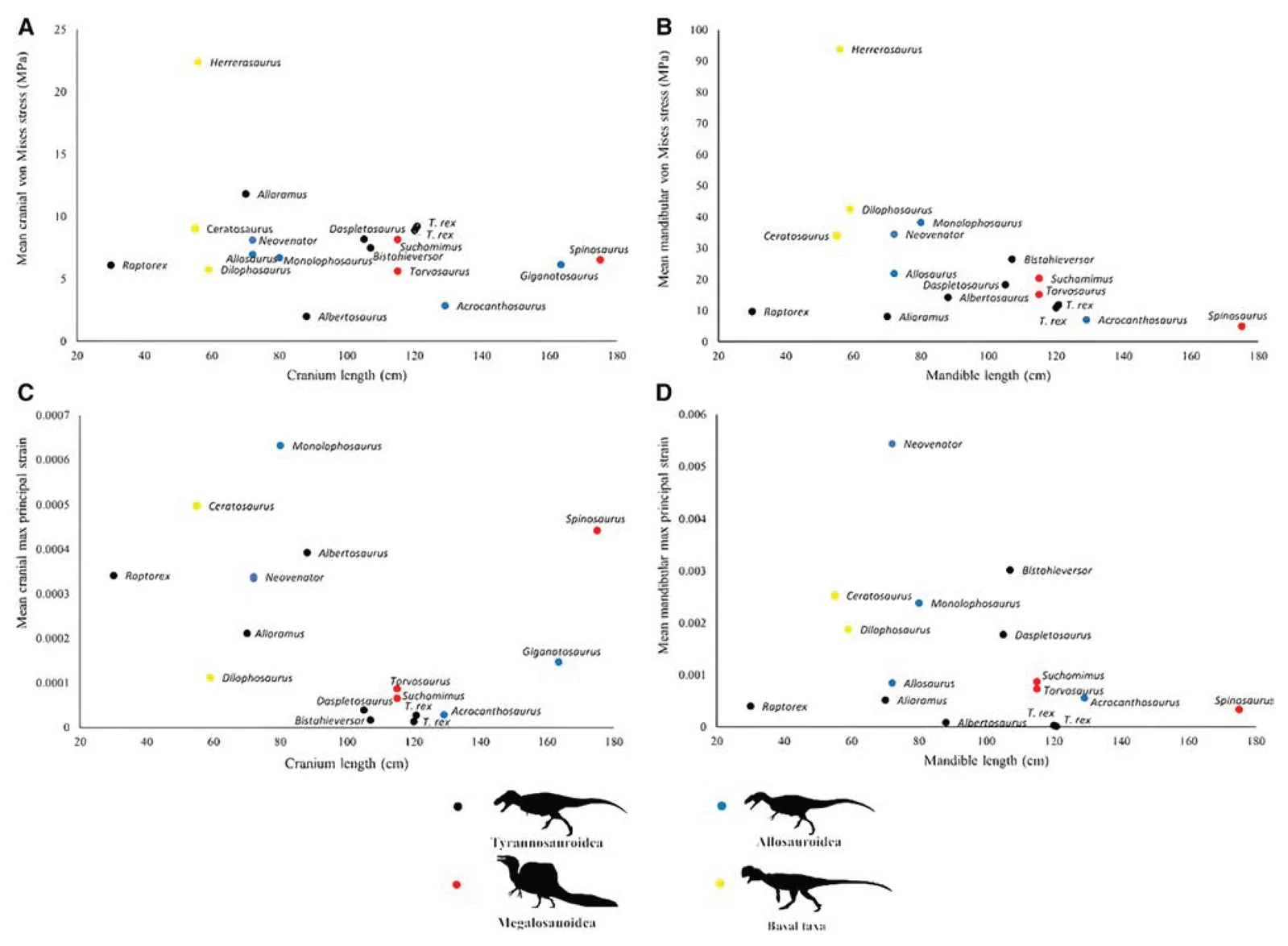
Theropod skull lengths versus von Mises stresses at actual size Cranial lengths plotted against mesh-weighted mean cranial von Mises stresses (A), mean mandible von Mises stress (B), mean cranial maximum principal strain (C), and mean mandible principal strain for each theropod dinosaur at actual size (D). *Herrerasaurus* is not included in (C) and (D) due to its high values. Skull lengths in cm. Note that strain is a unitless quantity. Theropod silhouettes from PhyloPic by Scott Hartman.

Mean maximum principal strain was calculated for the crania and mandibles (Figure 3). Mean maximum principal strain was highest in *Herrerasaurus*, with *Monolophosaurus, Ceratosaurus*, and *Spinosaurus* also showing elevated strain. Larger theropods (*Suchomimus, Acrocanthosaurus*, and *Giganotosaurus*) generally experienced lower strain, but no clear size-related trend was observed.

Calculating a linear regression with all skull lengths (x) and cranial von Mises stresses (y) yielded a *p* value of 0.3305, whereas the *p* value when tyrannosauroids were excluded yielded a *p* value of 0.2596, both indicating a weak relationship between the two. A linear regression analysis of the crania reveals a coefficient of determination value of R^2^ = 0.0581. Phylogenetically corrected regression analysis incorporating skull lengths and cranial von Mises stresses gave a *p* value of 0.5227, confirming low significance.

### Mandibles at actual size

Mandibles exhibited higher stresses than crania, with stress concentrated near the mandibular fenestrae, especially in *Dilophosaurus* and *Suchomimus*—likely due to the presence of wrapping pterygoid muscles. In allosauroids (e.g., *Acrocanthosaurus*), stress was concentrated toward the anterior jaw, whereas in tyrannosauroids (e.g., *Daspletosaurus*), stress was more pronounced at the posterior end.

As in previous plots, *Herrerasaurus* experiences the highest values, with certain medium-sized theropods also exhibiting high mandibular strain, including *Neovenator. Bistahieversor* demonstrated the highest mandibular strain of the large-bodied theropods.

Calculating a linear regression of non-tyrannosauroid theropods with skull lengths (x) and mandibular von Mises stresses (y) yielded a *p* value of 0.0163, indicating a highly statistically significant relationship between skull length and mandible stress. When tyrannosauroids were included in the calculation, a *p* value of 0.0628 was obtained, weakening the relationship. Phylogenetically corrected regression analysis gave a *p* value of 0.184, indicating weak significance.

### Crania when scaled to equivalent size

**Figure 4:**
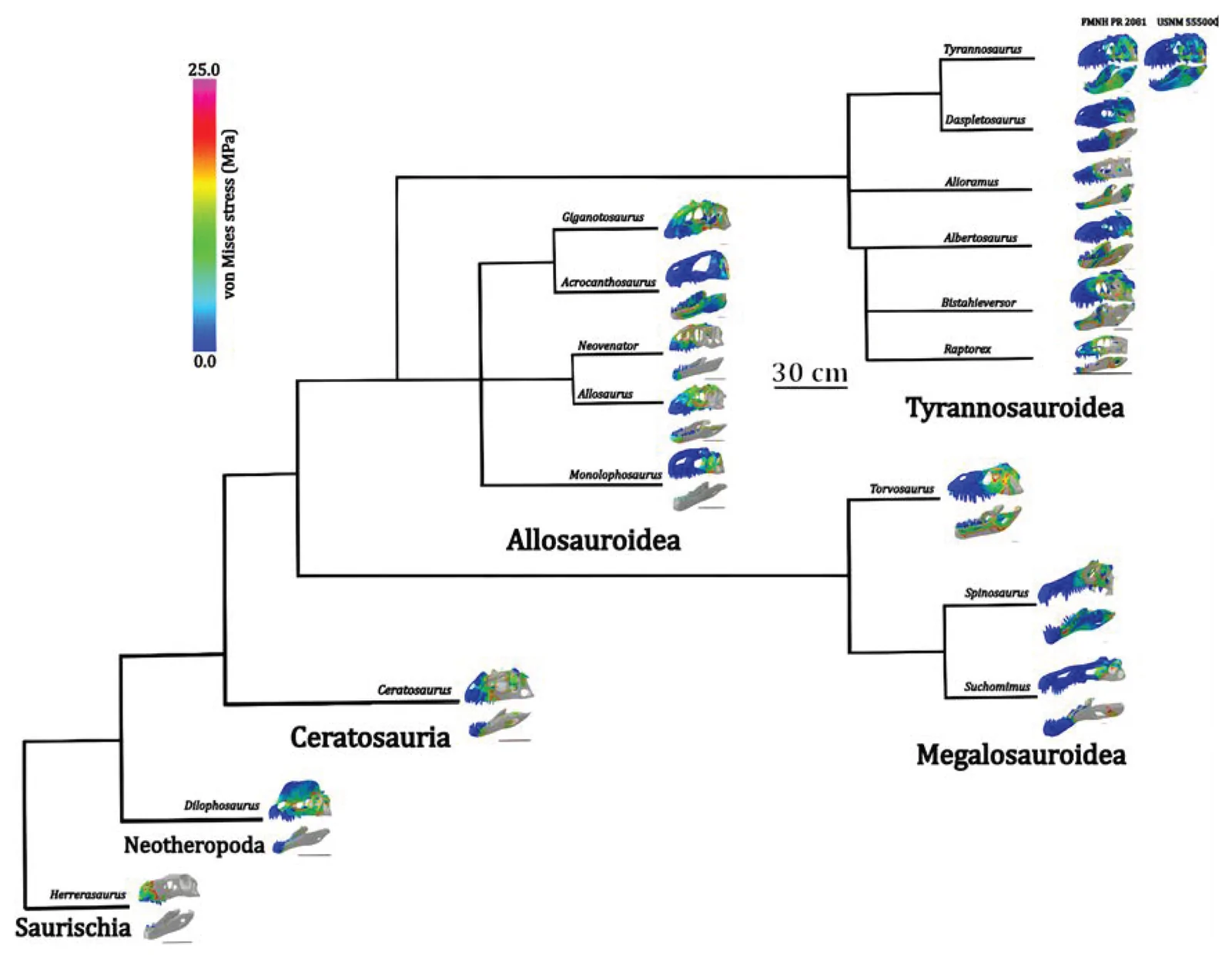
Finite element stress maps for all theropod skulls modeled when size corrected Gray regions indicate regions with the highest probabilities of structural failure. For larger stress maps of size-corrected skulls, see Figure S2.

These models assess how theropod skull shapes respond to feeding loads when skulls are scaled to the same equivalent size (equal muscle force to surface area ratio) (Figures 4 and S1).

*Herrerasaurus* and *Ceratosaurus*, two of the shortest skulls, demonstrate the highest stress values, whereas the large megalosauroids and allosauroids are generally the most stress resistant, especially *Acrocanthosaurus*. Tyrannosauroids generally become more stressed at smaller body size, such as *Raptorex* and *Alioramus*; *Tyrannosaurus* specimens exhibited low-stress levels, comparable to large megalosauroids and allosauroids. High stress remained concentrated near the quadrate and jugal bones, like the unscaled results.

**Figure 5:**
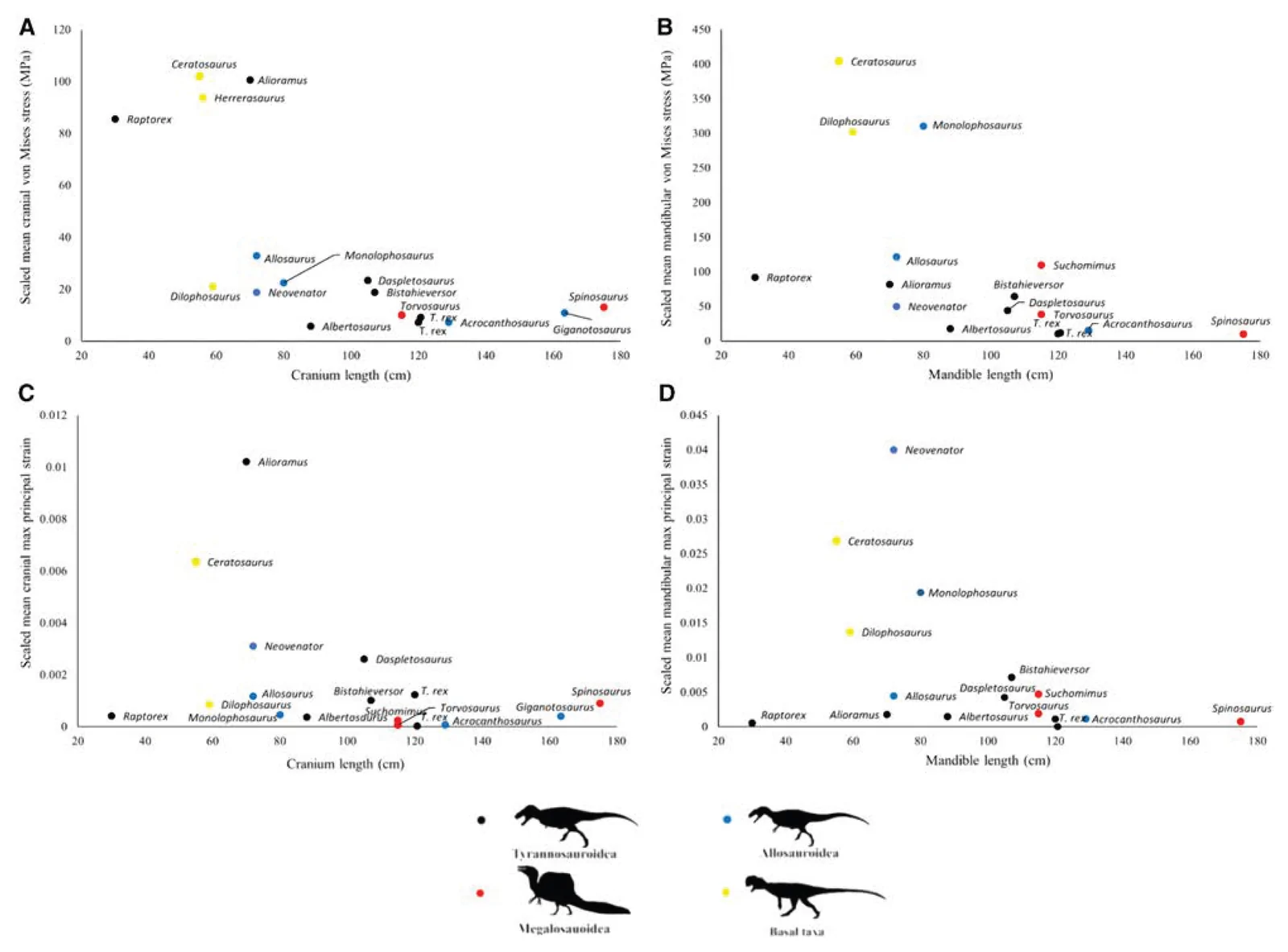
Theropod skull lengths versus von Mises stresses when size corrected Skull lengths plotted against calculated mesh-weighted mean cranial von Mises stress (A), mean mandibular von Mises stress (B), mean cranial maximum principal strain (C), and mean mandibular maximum principal strain for each theropod dinosaur cranium when size corrected (D). *Herrerasaurus* is not included in (B), (C), and (D) due to its high values. Skull lengths in cm. Theropod silhouettes from PhyloPic by Scott Hartman.

Cranial strain results for the muscle-force-scaled dataset revealed the highest strain in *Herrerasaurus, Ceratosaurus*, and *Neovenator* (Figures 5 and S2). Lowest strains were present in *Acrocanthosaurus* and *Suchomimus*. Strain results for the muscle-force-scaled mandibles yielded the highest strains in *Herrerasaurus* and *Neovenator*. The lowest values were reported in *Spinosaurus* and *Acrocanthosaurus*, two of the largest taxa in the dataset.

Calculating a linear regression with skull lengths (x) and cranial von Mises stresses (y) for the scaled muscle force dataset yielded a *p* value of 0.1981, whereas the *p* value when tyrannosauroids were excluded resulted in 0.2976, both indicating weak significance. Phylogenetically corrected regression analysis gives us a *p* value of 0.4376, confirming low statistical significance.

### Mandibles when scaled to equivalent size

Scaling to the muscle force to surface area ratio of *Tyrannosaurus* resulted in structural failure in the mandibles of smaller- and medium-sized taxa, including *Monolophosaurus, Neovenator, Allosaurus, Ceratosaurus*, and *Herrerasaurus*. Smaller tyrannosaurids (*Raptorex* and *Alioramus*) also experienced high stress. Larger non-tyrannosauroid tetanurans (*Spinosaurus* and *Acrocanthosaurus*) showed more even stress distribution along the mandible. *Dilophosaurus* exhibited high stress at the posterior, decreasing toward the anterior.

Consistent with other datasets, *Herrerasaurus* exhibited the highest mandibular strain, followed by *Neovenator*. Among large theropods, *Bistahieversor* demonstrated the highest mandibular strain, potentially due to 3D model reconstruction. *Dilophosaurus* also showed elevated strain.

Calculating a linear regression with skull lengths (x) and mandibular von Mises stresses (y) for the scaled muscle force dataset yielded a *p* value of 0.2411 and, when tyrannosauroids were excluded, a *p* value of 0.3046, both indicating weak significance. Phylogenetically corrected regression analysis gives a *p* value of 0.494, confirming low significance.

## Discussion

Non-avian theropod dinosaurs represent a classic example of Cope’s rule, exhibiting a macroevolutionary trend toward increasing body size through time. This study presents the largest 3D dataset of carnivorous theropod skulls analyzed using finite element analysis (FEA) to explore cranial and mandibular stress under feeding-related loads. Our findings are the first to document biomechanical performance across a broad phylogenetic and size range of theropods, revealing distinct functional patterns associated with body size evolution.

The greatest cranial stress resistance is observed in large-bodied non-tyrannosauroid theropods, particularly megalosauroids and allosauroids such as *Spinosaurus* and *Acrocanthosaurus*. These taxa exhibit relatively low von Mises stress during simulated feeding, especially in the cranium. In contrast, large tyrannosauroids display elevated cranial stress levels, largely attributable to their expanded posterior skull regions, which accommodate more adductor musculature and generate correspondingly higher bite forces. Despite this, stress magnitudes in all taxa remain within biomechanically tolerable limits.

These results reveal two divergent functional strategies among large-bodied theropods: low stress, lower-power strategy—typified by allosauroids, megalosauroids, and basal taxa, whose slenderer skulls and more distributed muscle loading reduce peak stresses; high-stress, high-power strategy—represented by tyrannosaurids, whose posteriorly expanded crania allow for greater adductor muscle mass and stronger, bone-crushing bites.

Our hypotheses are partially supported: the null hypothesis (1) is partially falsified, whereas hypotheses (2) and (3) are upheld. Large non-tyrannosauroids indeed exhibit lower stress magnitudes compared with similarly sized tyrannosauroids, and smaller theropods, once size-corrected, tend to approach stress thresholds that might increase failure risk.

Functional divergence likely reflects ecological specialization. Tyrannosaurids, including *Tyrannosaurus rex*, combined stiff, akinetic skulls with high bite forces (up to ~60,000 N) facilitated by fused nasals and widely spaced quadrates, allowing efficient force transmission during predation.^48–50^ These features point toward possible ambush predation strategies, consistent with low rotational inertia, limited cursorial ability, and fossil evidence of active predation.^24,51,52^ Subadult and slender-skulled forms such as alioramins may have pursued more agile prey.^53,54^ When scaled to actual size, tyrannosauroids continue to show high biting stress, yet their cranial architecture efficiently manages these loads. Their distinctive traits—fused cranial elements, robust mandibles, and posteriorly expanded skulls—distinguish them biomechanically from other large theropods. In contrast, large tetanurans like *Allosaurus* and *Monolophosaurus* likely adopted more generalized feeding strategies involving lower bite forces and slashing bites, aided by wider gapes, cranial flexibility, and avian-like prey manipulation.^55,56^ The elevated skull stress observed in *Herrerasaurus* is notable given its phylogenetic position. As a basal taxon, it retains plesiomorphic features that may predispose its skull to higher mechanical stress under simulated biting loads. Although model reconstructions were conservative and guided by phylogenetic bracketing, some exaggeration of stress magnitudes is possible due to those reconstructions, particularly in regions influencing stress distribution.^35^

Mandibular stress trends differ between lineages. Although tyrannosauroid mandibles show increasing stress with body size, large tetanuran mandibles show a statistically significant trend toward reduced stress. This may reflect differences in prey acquisition tactics: stiff, puncture-pull biting in tyrannosaurids versus slashing or tearing in allosauroids, where broader gape mechanics and kinetic cranial elements may have modulated stress distributions.

Medium-sized theropods (e.g., small allosauroids) experience skull stresses comparable to their larger relatives, implying over-lapping feeding strategies or targeting of similar prey resources. The apparent ecological success of tyrannosauroids and megalosauroids may partly reflect biomechanical performance advantages at large body sizes, potentially enabling them to displace or outcompete mid-sized clades such as abelisauroids—a pattern reflected in the relative scarcity of mid-sized carnivores in some ecosystems.^57^ Spinosaurids, in contrast, are often interpreted as semi-aquatic piscivores.^58–62^ Their elongated rostra and reduced antorbital fenestrae contribute to increased torsional resistance despite low bite forces relative to other large theropods.^63^ Although often associated with aquatic prey, fossil evidence, e.g., gut contents containing pterosaurs and iguanodontids, suggests a broader dietary repertoire.^64–66^ Their cranial morphology thus reflects both specialization and flexibility.

Overall, large body size conferred biomechanical advantages across theropod lineages by enhancing muscle mass and bite force, enabling broader prey spectra. However, skull shape also constrained performance. Taxa with short, slender skulls underperformed when size-corrected, indicating shape-function interactions. Similar conclusions were drawn by Ma et al.,^31^ who identified a trend of jaw strengthening across theropod clades.

Morphologically, phylogeny appears to drive skull form more than function at broad scales,^67^ but our results suggest a tighter link between function, form, and ecology at finer phylogenetic resolutions. Similarly, Lautenschlager^68^ found that orbit shape, e.g., elliptical or keyhole shaped, enhances stress dissipation, particularly in large-bodied taxa—reinforcing the idea that skull design evolved under complex, size-dependent constraints. Mandibular and cranial elements reveal differing biomechanical patterns, likely due to their distinct functional roles.

The vertebrate mandible is assumed to be more closely adapted for feeding function, whereas the cranium is subject to multiple constraints^69^; hence, our cranial and mandibular results do not demonstrate identical trends. The mandible, primarily adapted for feeding, shows a clearer relationship with body size and stress. In contrast, the cranium—subject to multiple selective pressures, including sensory and display functions—shows weaker size-stress correlations. These findings support the view that cranial morphology in theropods evolved under multi-functional constraints.

Cranial ornamentation, such as crests and horns (e.g., in *Dilophosaurus* and *Ceratosaurus*), appears to have limited impact on feeding performance. Stress values in ornamented taxa are comparable to those of similarly sized, unornamented theropods. Although Johnson-Ransom et al.^41^ suggested a potential stress-reducing role for the crest in *Proceratosaurus*, this may represent a lineage-specific adaptation rather than a general functional pattern.

## Conclusion

Large theropods evolved divergent biomechanical strategies as they attained gigantism, reflecting distinct ecological and evolutionary pressures. Tyrannosaurids, in particular, developed deep, powerfully muscled skulls that tolerated high levels of feeding-induced stress—an adaptation consistent with bone-crunching predation and the exploitation of giant prey. This shift toward maximizing bite force may have been driven by the unique demands of Late Cretaceous ecosystems, including competition with smaller theropods and giant crocodyliforms, as well as the emergence of heavily armored herbivores like ankylosaurs. In contrast, other giant theropods—such as allosauroids and megalosauroids—retained more generalized cranial architectures that experienced lower stress, suggesting functional strategies optimized for less-force-intensive feeding. Although cranial biomechanics was not the only factor shaping body size evolution in Theropoda, our results indicate that feeding performance played a key role in determining how different lineages achieved—and sustained—ecological dominance. These contrasting strategies highlight multiple evolutionary pathways to mega-carnivory and underscore the deep functional diversity that underpinned the success of large-bodied predatory dinosaurs.

## Resource Availability

### Lead contact

Further information and requests for resources should be directed to and will be fulfilled by the lead contact, Andre J. Rowe (andre.rowe@bristol.ac.uk).

### Materials availability

This study did not generate new reagents.

## Star★Methods

### Key Resources Table

**Table:** 

Specimen name	Specimen number	Skull length (cm)	Body mass estimate (kg)	Body mass source
*Herrerasaurus ischigualastensis*	PVSJ 407	56	350	Paul^76^
*Dilophosaurus wetherilli*	UCMP 77270	59	400	Paul^76^
*Ceratosaurus nasicornis*	BYUVP 12893	55	418	Therrien and Henderson^8^
*Monolophosaurus jiangi*	IVPP 84019	80	475	Paul^76^
*Allosaurus jimmadseni*	MOR 693	72	1,500	Bates et al.^77^
*Neovenator salerii*	MIWG.6348	72	1,000	Paul^76^
*Acrocanthosaurus atokensis*	NCSM 14345	129	2,551.25	Snively et al.^51^
*Giganotosaurus carolinii*	MUCPv-Ch1	163.4	3,540.64	Snively et al.^51^
*Torvosaurus gurneyi*	BYUVP 2002–2014, 2016–2018, 4882–4884, 4860, 4890, 4998, 5110, 5286, 9120–9122, 9249	115	1,650	Hendrickx and Mateus^78^
*Spinosaurus aegyptiacus*	FSAC-KK 11888	175	7,400	Sereno et al.^62^
*Suchomimus tenerensis*	MNN GDF501	119	3,816.1	Seebacher^79^
*Raptorex kriegsteini*	LH PV18	30	65	Sereno et al.^80^
*Bistahieversor sealeyi*	NMMNH P-27469	107	3,300	Molina-Pérez et al.^81^
*Albertosaurus sarcophagus*	TMP 1981.010.0001	88	1,685	Christiansen and Fariña^7^
*Alioramus altai*	IGM 100/1844	70	369	Brusatte et al.^82^
*Daspletosaurus torosus*	FMNH PR308	105	2,700	Christiansen and Farina^7^
*Tyrannosaurus rex*	USNM 555000	120	6,216	Campione et al.^83^
*Tyrannosaurus rex*	FMNH PR 2081	127.5	7,377	Campione et al.^83^
Body masses were referenced from the literature and skull lengths were measured in MeshLab 2020.06.				

### Experimental Model and Study Participant Details

#### Taxonomic Sampling

##### Megalosauroidea

Megalosauroidea (also referred to as Spinosauroidea) is a clade of tetanuran theropods that lived from the Middle Jurassic to the Late Cretaceous and may be paraphyletic with respect to Allosauroidea.^70^ They represent one of the first major radiations of giant theropod dinosaurs.^71^ The possibly semi-aquatic *Spinosaurus* may have been the longest bipedal dinosaur, with some estimates exceeding 14 m.^62^ There is evidence for body size increase through time within the clade: basal taxa from the Early Jurassic were smaller than later Middle Jurassic forms.^72^ The largest members—*Spinosaurus* and *Suchomimus*—are from North Africa,^73,74^ but megalosauroids are also known from North America, South America, and possibly Asia.^75^ The taxa included in this study were *Torvosaurus, Spinosaurus*, and *Suchomimus* (Table 1).

##### Allosauroidea

Allosauroids are tetanuran theropods whose earliest known representative is *Shidaisaurus* from Aalenian-aged deposits (~174 Ma) in Middle Jurassic China.^39^ Among the latest-surviving taxa is *Carcharodontosaurus* from the Cenomanian of North Africa (~100 Ma), which may have coexisted and competed with *Spinosaurus*. Another large allosauroid, *Giganotosaurus*, lived in Argentina during the same interval and likely served as a generalized predator.^84^ Allosauroids had a wide geographic range across both hemispheres during the Jurassic and Early Cretaceous but were later displaced by tyrannosaurids in the Late Cretaceous.^1^ Distinctive features include relatively long forelimbs, large orbits, and narrow skulls. Their teeth and skull morphology suggest a slicing feeding style, similar to Komodo dragons,^82^ contrasting with the bone-crushing bites of tyrannosaurids, which are more akin to those of extant crocodilians.^49^ This study included *Monolophosaurus, Allosaurus, Neovenator, Giganotosaurus*, and *Acrocanthosaurus* (Figure 1 and Table 1).

##### Tyrannosauroidea

Tyrannosauroids are coelurosaurian theropods that first appeared in the Oxfordian of the Middle Jurassic (~160 Ma) in Asia and later dispersed to North America.^45^ Large, derived tyrannosaurids such as *Tyrannosaurus* exhibit broad skulls, reduced forelimbs, and long legs relative to body size. Mass estimates for *Tyrannosaurus* are the highest for any known theropod, ranging from 6,000 to 9,500 kg.^8,85^ Some lineages, such as the alioramins, retained gracile morphologies into adulthood—potentially reflecting ecological differentiation and separation of resource use in Late Cretaceous ecosystems. Such divergence may have reduced interspecific competition and enabled the coexistence of multiple apex predators within a single faunal assemblage.^15^ Owing to their excellent fossil record and public interest, tyrannosauroids are among the most studied theropods, particularly in terms of feeding biomechanics.^29,32,41,48,49,54^ Nevertheless, few studies have compared these biomechanical traits in a broad macroevolutionary context. The taxa sampled here were *Raptorex, Bistahieversor, Albertosaurus, Alioramus, Daspletosaurus*, and two specimens of *Tyrannosaurus* (Figure 1 and Table 1).

### Method Details

#### 3D imaging and model generation

Skull lengths and 3D models were compiled from the literature and specimen scanning to assess potential relationships between size and cranial stress (Tables 1 and S1). Models were generated using either CT scanning or structured-light surface scanning^35^ (Table S2), depending on the specimen’s size and availability. Surface scanning was preferred for mounted skeletons or large specimens, which are often inaccessible for CT scanning.

Models were derived from original fossils and high-quality casts. Some were composites due to missing anatomical elements, which were reconstructed using closely related taxa. For instance, missing cranial bones in the *Suchomimus* model were based on the Brazilian spinosaurid *Irritator*,^86^ and missing elements in *Torvosaurus* were modeled after related megalosauroids.

Scanning was performed at multiple institutions: *Allosaurus* was CT scanned from original material; *Spinosaurus* and *Giganotosaurus* were CT scanned from casts. Surface scans were performed at the Dinosaur Discovery Museum (DDM) using elevated platforms for mounted specimens (*Suchomimus, Torvosaurus, Acrocanthosaurus, Ceratosaurus, Herrerasaurus*), at the Milwaukee Public Museum for *Monolophosaurus*, and at the Science Museum of Minnesota for *Dilophosaurus*. The *Neovenator* model was based on a museum sculpture, with missing regions informed by *Allosaurus* morphology (M. Munt, pers. comm., 2021). Despite varying model sources, interspecific differences were expected to outweigh any cast-specific discrepancies.

#### Model processing and editing

Surface scans were processed in Artec Studio 14 Professional, using real-time fusion at 7–8 frames/sec. Crania and mandibles were scanned separately, aligned manually, and merged into single STL files. Frames with high error, stray pixels, and non-cranial elements (e.g., cervical vertebrae) were removed. Global Registration and Sharp Fusion were applied to preserve detail and create watertight, polygonal models. Floating pixels and holes were corrected using in-built filters.

The *Dilophosaurus* model, based on a half-skull resin cast, was mirrored and reconstructed in Blender 2.82, then cleaned of intersecting triangles in Geomagic Studio 12. Final edits, including hole filling and mesh correction, were also completed in Geomagic Studio 12. At this stage, model surface area, triangle count, element count, and volume were measured and standardized across specimens to reduce potential analytical bias (Tables S1 and S2).

Several specimens posed reconstruction challenges. *Acrocanthosaurus* was part of a full skeletal mount on public display at the DDM. The skull was centrally positioned and elevated, necessitating ladder access and scanning at awkward angles with limited maneuverability. As a result, aspects of the cranial surface exhibit areas of low-resolution and accuracy due to restricted scanner coverage, which appears in the finalized 3D model. The skull roof of *Daspletosaurus* was similarly difficult to interpret due to complex surface topography and elevation. In *Bistahieversor*, fine details of the parietal region were reconstructed by referencing *Tyrannosaurus rex* (FMNH PR 2081).

The internal anatomy of *Torvosaurus* and *Suchomimus* was reconstructed using anatomical comparisons with *Spinosaurus* due to a lack of preserved internal cranial features. While general morphology was retained, the endocranial surfaces and internal structures may be simplified or exhibit ‘blocky’ geometries.

Several specimens, including *Acrocanthosaurus, Suchomimus, Torvosaurus, Herrerasaurus, Ceratosaurus*, and *Monolophosaurus* were scanned while articulated with cervical vertebrae as part of full skeletal mounts. To isolate the cranium, the anterior cervical vertebrae (C1–C2 in nearly all models) were digitally removed post hoc, and the occipital region—including the occipital condyle, basioccipital, and posterior portions of the parietals and squamosals—was reconstructed based on preserved morphology and comparisons with related taxa.

The *Alioramus* skull was disarticulated and previously CT scanned as approximately 30 individual elements, each provided digitally as separate files. These were imported and aligned in Blender 2.82 through careful manual articulation, with anatomical orientation guided by osteological landmarks and comparison with related tyrannosaurids.^54^

#### Finite element analysis (FEA)

Meshing was conducted in HyperMesh (Altair), using tetrahedral elements with 10 mm size and optimized quality settings. Each model was assigned material properties based on crocodilian bone (Young’s modulus = 15,000 MPa; Poisson’s ratio = 0.29) to approximate dinosaur bone.^87–89^ Dentine properties were not modeled, as they contribute minimally to stress distribution in comparative analyses.^90^

Constraint nodes were placed at the quadrate (crania) and articular (mandibles) to prevent rigid body motion, and additional constraints were applied to the premaxillary teeth to simulate prey contact.^91^ Muscle forces were estimated based on attachment sites from the literature^32,54,61^ (Figures S3–S5); forces were scaled from *Tyrannosaurus* (FMNH PR 2081) using the subtemporal fenestra method.^92^ Muscle cross-sectional area was measured in ImageJ and multiplied by an isometric muscle stress of 31.5 N/cm^2^ (Figure S6 and Table S3).

To normalize body size and isolate the effects of skull shape, we scaled each model’s muscle force relative to its surface area (Table S4). FEA simulations were rerun at both actual and scaled sizes, and von Mises stress and maximum principal strain were visualized using 3D stress maps with a 0–25 MPa stress range. Skull and mandible stress values were analyzed separately, given the fragility of mandibles.

#### Limitations of the Study

One limitation of this study pertains to the anatomical reconstructions of surface scanned skulls. As this scanning method only captures the external geometries of objects, we must use figures from the literature and existing CT data to reconstruct the internal anatomy of surface scanned-object files. CT-derived 3D models are likely more reflective of biological reality due to the preservation of air-filled spaces in the diapsid skull, though surface scanning was previously tested and found to be reliable in comparative studies in Rowe and Rayfield,^35^ particularly when assessing overall patterns of stress and strain. Additionally, several cranial models were affected by scanning constraints or limited fossil preservation, which may introduce minor inaccuracies. For example, the *Acrocanthosaurus* model was derived from a mounted specimen scanned under difficult conditions, limiting surface fidelity in some regions.

While the finite element results are broadly consistent across lineages, some aspects of stress magnitude and distribution are influenced by reconstruction choices, including muscle origin and insertion sites. We based our muscle placements on previous reconstructions^32,54^ to produce conservative and repeatable estimates. However, alternative reconstructions exist; for example, those recently proposed by Johnson-Ransom et al.^41^ which reflect different interpretations of soft tissue anatomy. Although such differences are unlikely to overturn our main conclusions, future comparative FEA studies would be valuable to test how varying muscle topologies affect cranial stress patterns.

#### Data analysis

Mesh-weighted arithmetic mean (MWAM) von Mises stress values were calculated in RStudio^93^ to account for mesh resolution and element volume.^94–96^ Models with extensive editing, e.g., *Dilophosaurus*, were interpreted cautiously, as prior work suggests such models may yield elevated stress values.^35^ Final data were plotted against skull length to assess the relationship between size and stress (Figures 3, 5, S1, and S2).

### Quantification and Statistical Analysis

Statistical significance was tested using p-values and phylogenetic generalized least squares (PGLS) regression in RStudio with the ‘ape’ package.^97^ Regression outputs are presented in the Results section; N=18 and represents the total number of dinosaur taxa. No additional statistical tests were applied.^76–81,83^

### Additional Resources

No additional resources were used in this study.

## Supplementary Material

Supplemental information can be found online at https://doi.org/10.1016/j.cub.2025.06.051.

Supplementary files

## Figures and Tables

**Table 1:** Skull lengths and body mass estimates for each taxon ordered by phylogenetic position

REAGENT or RESOURCE	SOURCE	IDENTIFIER
Software and algorithms		
Abaqus/CAE	https://www.3ds.com/products/simulia/abaqus/cae/	Abaqus/CAE Version 6.14-1
Altair HyperMesh	https://altair.com/	HyperMesh Version 2021
Artec Studio Professional	https://www.artec3d.com/3d-software/artec-studio/	Artec Studio Professional Version 14
Avizo Lite	https://www.thermofisher.com/uk/	Avizo Lite Version 9.5
Blender	https://www.blender.org/	Blender Version 2.82
Geomagic Studio	https://www.3dsystems.com/software/	Geomagic Studio Version 12
MeshLab	https://www.meshlab.net/	MeshLab 2020.06
RStudio	RStudio Team https://www.rstudio.com/	RStudio Version 2023.06.1

## Data Availability

Dinosaur skull STL files are available with permission from their respective institutions where scanning took place. No original code was generated in this study. Any additional information required to reanalyze the data reported in this paper is available from the lead contact upon request.

## Highlights

- Large theropods evolved distinct biomechanical strategies for feeding
- Tyrannosaurids prioritized bite force, leading to higher skull stress
- Megalosauroids and allosauroids maintained low-stress skull designs
- Skull stress patterns reflect ecological divergence in carnivorous dinosaurs

## In brief

Rowe and Rayfield reveal two evolutionary paths to theropod dominance: tyrannosaurids crushed prey with high-stress skulls, whereas other clades sliced with low-stress bites. These biomechanical extremes underscore how feeding power shaped Mesozoic ecosystems.
