# Supplementary material for: Carnivorous dinosaur lineages adopt different skull performances at gigantic size
Source: Curr Biol. Author manuscript; Available in PMC 2026 Oct 23. (PMC7619621; doi:10.1016/j.cub.2025.06.051)
Supplement: Supplementary file 1 [file EMS218226-supplement-Supplementary_files.zip › 1-s2.0-S0960982225008115-mmc1.pdf]

Current Biology, Volume *35*

Supplemental Information

Carnivorous dinosaur lineages adopt different skull performances at gigantic size

Andre J. Rowe and Emily J. Rayfield

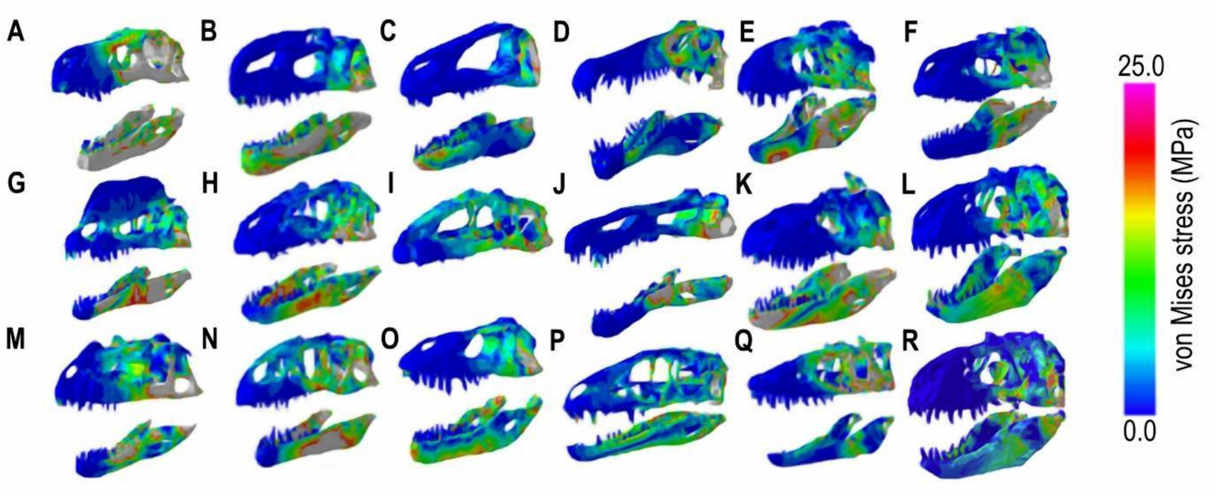

Figure S1. Larger FE models of the actual size results. Related to Figure 2.

(A) *Herrerasaurus*, (B) *Monolophosaurus*, (C) *Acrocanthosaurus*, (D) *Spinosaurus*, (E) *Bistahieversor*, (F) *Daspletosaurus*, (G) *Dilophosaurus*, (H) *Allosaurus,* (I) *Giganotosaurus,* (J) *Suchomimus,* (K) *Albertosaurus,* (L) *Tyrannosaurus* (USNM 555000), (M) *Ceratosaurus*, (N) *Neovenator*, (O) *Torvosaurus,* (P) *Raptorex*, (Q) *Alioramus*, (R) *Tyrannosaurus* (FMNH PR 2081).

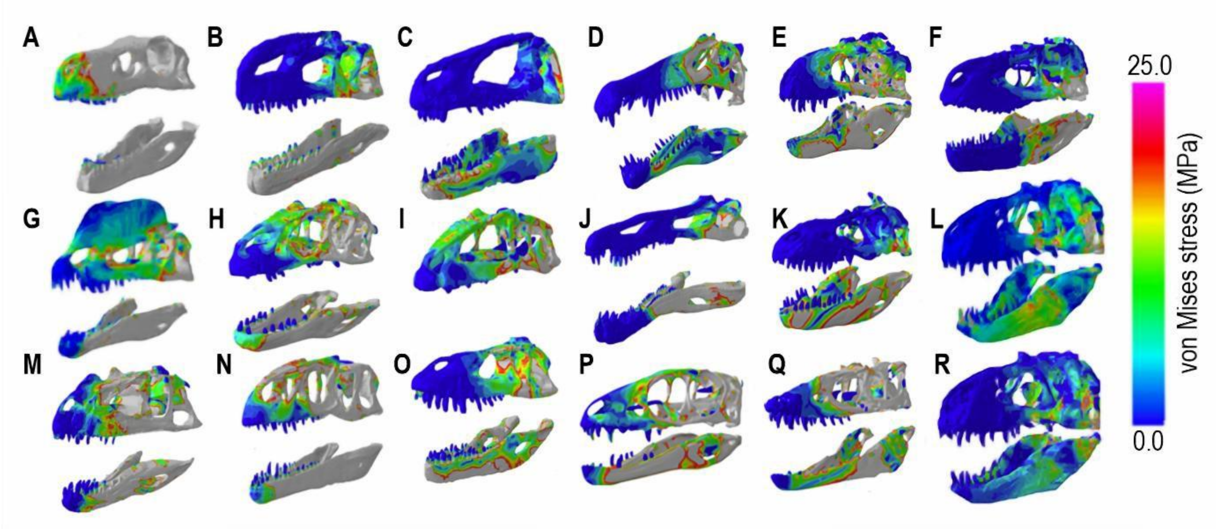

Figure S2. Larger FE models of the size corrected results. Related to Figure 4.

(A) *Herrerasaurus*, (B) *Monolophosaurus*, (C) *Acrocanthosaurus*, (D) *Spinosaurus*, (E) *Bistahieversor*, (F) *Daspletosaurus*, (G) *Dilophosaurus*, (H) *Allosaurus,* (I)

Giganotosaurus, (J) *Suchomimus,* (K) *Albertosaurus,* (L) *Tyrannosaurus* (USNM 555000), (M) *Ceratosaurus*, (N) *Neovenator*, (O) *Torvosaurus,* (P) *Raptorex*, (Q) *Alioramus*, (R) *Tyrannosaurus* (FMNH PR 2081).

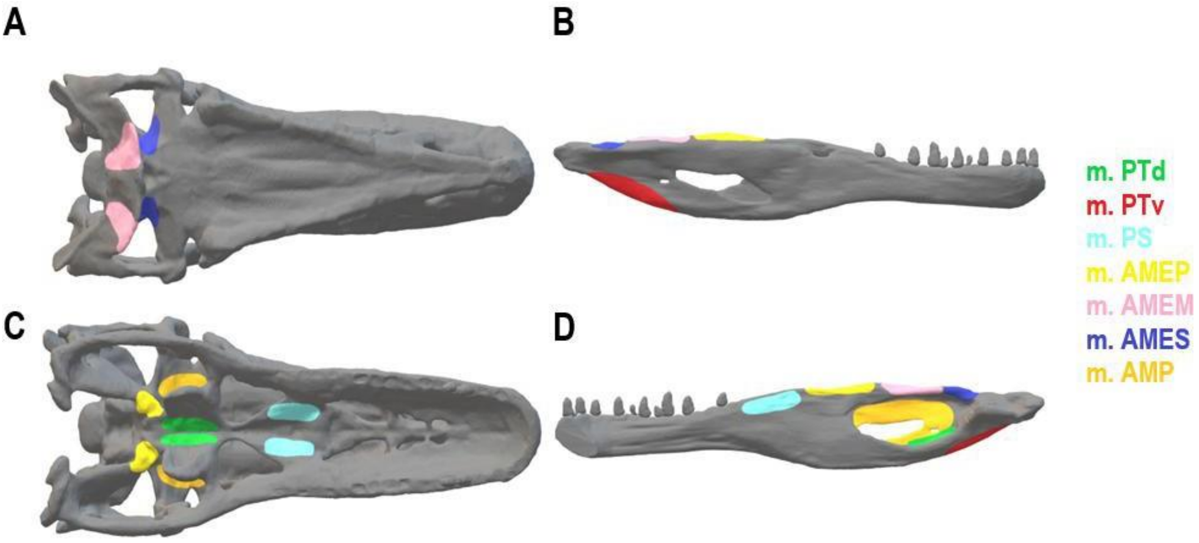

Figure S3. Locations of muscle insertions for *Allosaurus jimmadseni* (MOR 693) to represent muscle mapping for allosauroids in this study. Related to STAR*Methods.

(A) Cranium in dorsal view, (B) mandible in lateral view, (C) cranium in ventral view, and (D) mandible in medial view. Muscle abbreviations: m. PTd, M. pterygoideus dorsalis; m. PTv, M. pterygoideus ventralis; m. PS, M. pseudotemporalis complex; m. AMEP, M. adductor mandibulae externus profundus; m. AMEM, Musculus adductor mandibulae externus medialis; m. AMES, M. adductor mandibulae externus superficialis; m. AMP, M. adductor mandibulae posterior.

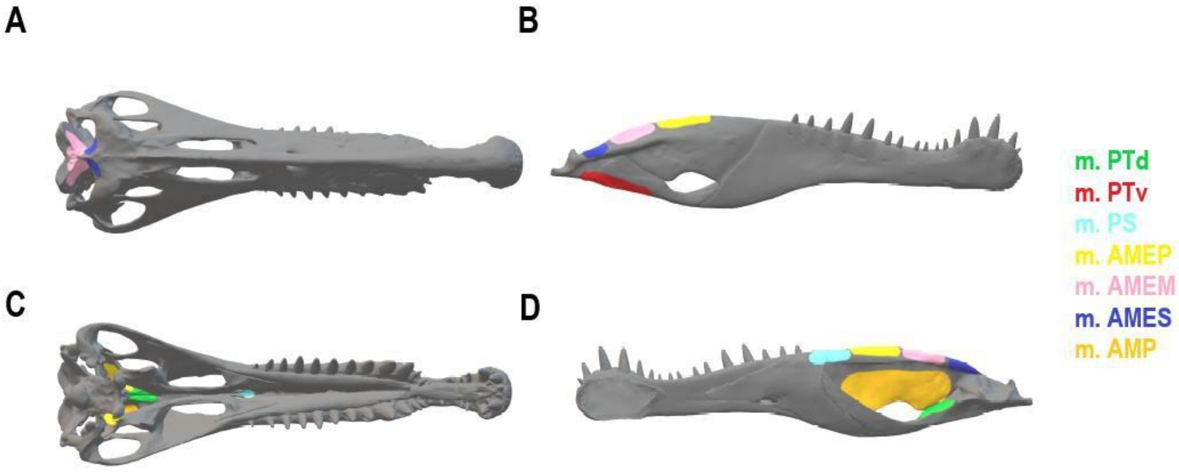

Figure S4. Locations of muscle insertions for *Spinosaurus aegyptiacus* (FSAC-KK 11888) to represent muscle mapping for megalosauroids in this study. Related to STAR*Methods.

(A) Cranium in dorsal view, (B) mandible in lateral view, (C) cranium in ventral view, and (D) mandible in medial view.

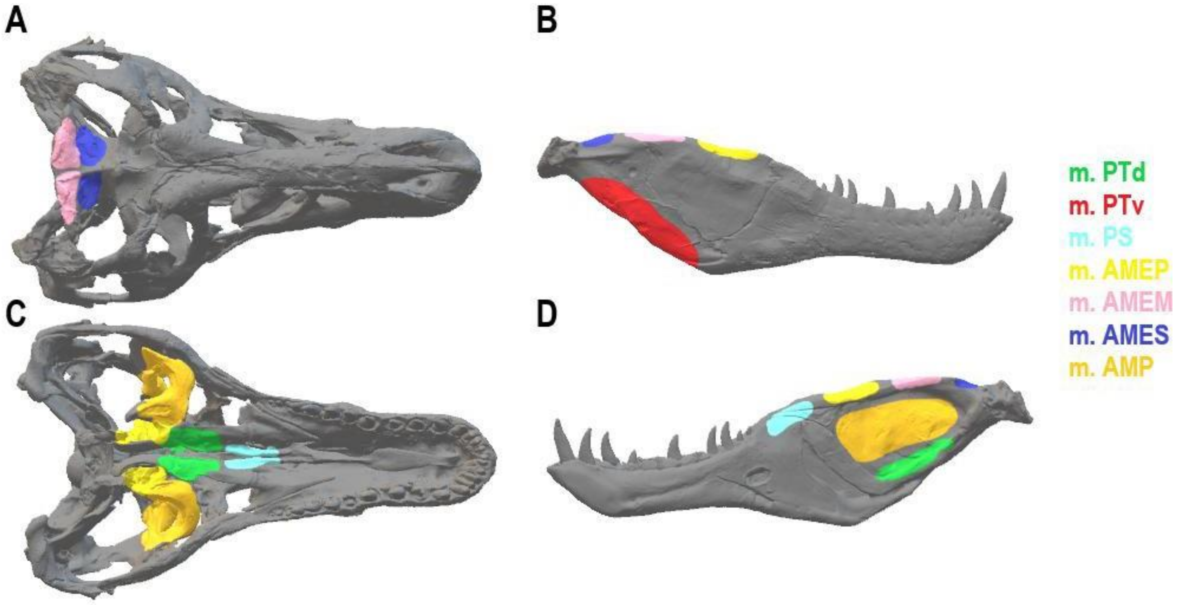

Figure S5. Locations of muscle insertions for *Tyrannosaurus rex* (FMNH PR 2081) to represent muscle mapping for tyrannosauroids in this study. Related to STAR*Methods.

(A) Cranium in dorsal view, (B) mandible in lateral view, (C) cranium in ventral view, and (D) mandible in medial view.

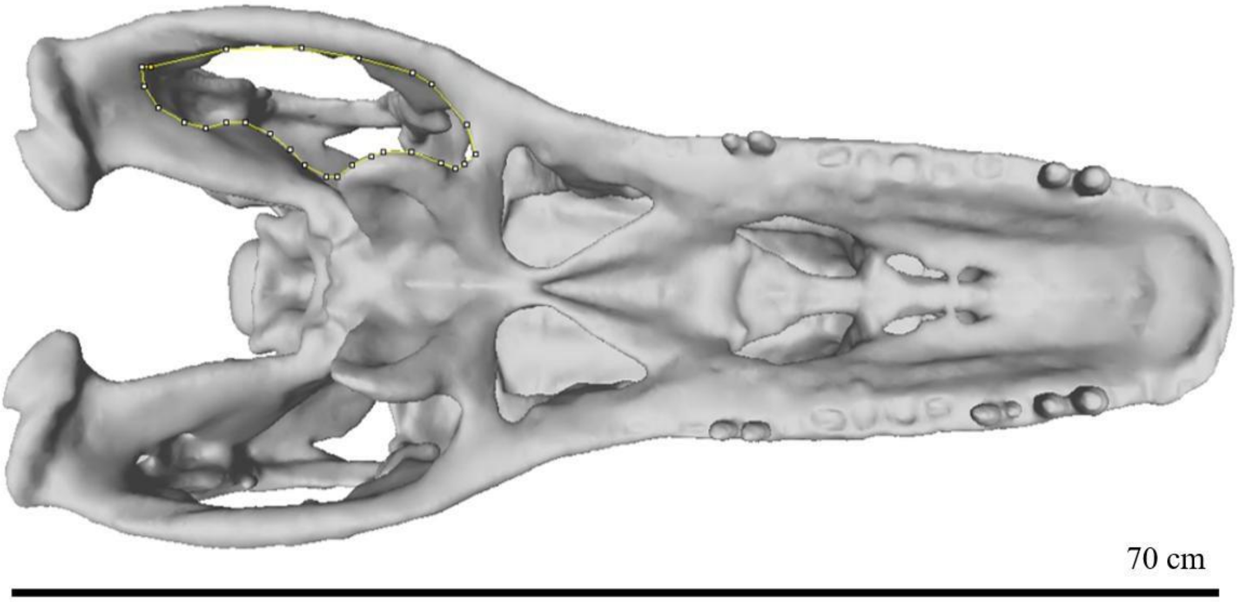

Figure S6. ***Allosaurus jimmadseni*** **(MOR 693) cranium in ventral view, with one adductor chamber measured in ImageJ (Fiji). Related to STAR*Methods.**

Adductor chamber measurements were multiplied by isometric muscle tension 31.5 N/cm^2 to calculate muscle force components applied in Abaqus/CAE 6.14-1.

Specimen	Cranial triangle count	Mandibular triangle count	Cranial element count	Mandibular element count	Cranial volume (mm³)	Mandibular volume (mm³)
Herrerasaurus ischigualastensis	350,006	315,016	525,009	472,524	174,985	157,506
Dilophosaurus wetherilli	349,972	299,972	524,958	449,958	174,974	149,984
Ceratosaurus nasicornis	398,798	314,918	598,197	472,377	199,391	157,459
Monolophosaurus jiangi	369,856	309,978	554,784	464,967	184,912	154,991
Allosaurus jimmadseni	155,102	114,838	232,613	172,257	77,493	57,411
Neovenator salerii	599,992	499,990	899,988	749,985	299,974	249,997
Acrocanthosaurus atokensis	400,014	350,026	600,021	525,039	200,001	175,015
Giganotosaurus carolinii	97,534	N/A	146,301	N/A	248,741	N/A
Torvosaurus gurneyi	499,234	400,066	748,851	600,099	249,613	200,031

Spinosaurus aegyptiacus	366,500	229,448	549,750	344,172	285,040	214,716
Suchomimus tenerensis	499,980	400,008	749,970	600,012	249,988	200,000
Raptorex kriegsteini	289,478	204,770	434,217	307,155	144,711	102,385
Bistahieversor sealeyi	923,682	529,002	1,385,523	793,503	461,821	264,485
Albertosaurus sarcophagus	651,876	317,422	977,814	476,133	325,886	158,705
Alioramus altai	465,994	38,102	698,991	57,153	227,012	19,045
Daspletosaurus torosus	386,920	467,596	580,380	956,394	616,518	318,802
Tyrannosaurus rex (USNM 555000)	1,270,954	711,910	1,906,431	1,067,865	635,435	355,957
Tyrannosaurus rex (FMNH PR 2081)	1,284,114	860,746	1,926,171	1,291,119	642,017	430,375

Table S1. 3D model triangles, elements, and volume measurements (mm^3). Related to STAR*Methods.

All values were calculated in MeshLab 2020.06.

Specimen name	Cranial model surface area (mm^2)	Mandible model surface area (mm^2)	Replica of skull?
Herrerasaurus ischigualastensis	94382	51683	Yes
Dilophosaurus wetherilli	608045	184758	Yes
Ceratosaurus nasicornis	360584	182932	Yes
Monolophosaurus jiangi	551013	249787	Yes
Allosaurus jimmadseni	788710	306512	Yes
Neovenator salerii	567398	245641	No
Acrocanthosaurus atokensis	1545905	1169611	Yes
Giganotosaurus carolinii	1880562	N/A	Yes
Torvosaurus gurneyi	1815301	869156	Yes
Spinosaurus aegyptiacus	2257382	1575902	Yes
Suchomimus tenerensis	828898	677021	Yes

Raptorex kriegsteini	904346	49811	Yes
Bistahieversor sealeyi	1777871	885024	No
Albertosaurus sarcophagus	1382871	677021	Yes
Alioramus altai	457843	160486	No
Daspletosaurus torosus	1526939	803412	Yes
Tyrannosaurus rex (USNM 555000)	4600059	1921720	Yes
Tyrannosaurus rex (FMNH PR 2081)	4604695	1924344	No

Table S2. Surface area values (mm^2) for the cranial and mandibular models of each taxon and the replica status of each skull included. Related to STAR*Methods.

All values were calculated in MeshLab 2020.06.
