# Supplementary material for: Carnivorous dinosaur lineages adopt different skull performances at gigantic size
Source: Curr Biol. Author manuscript; Available in PMC 2026 Oct 23. (PMC7619621; doi:10.1016/j.cub.2025.06.051)
Supplement: Supplementary file 1 [file EMS218226-supplement-Supplementary_files.zip › 1-s2.0-S0960982225008115-mmc4.pdf]

Current Biology

Article

Carnivorous dinosaur lineages adopt different skull performances at gigantic size

Highlights

- Large theropods evolved distinct biomechanical strategies for feeding
- Tyrannosaurids prioritized bite force, leading to higher skull stress
- Megalosauroids and allosauroids maintained low-stress skull designs
- Skull stress patterns reflect ecological divergence in carnivorous dinosaurs

Authors

Andre J. Rowe, Emily J. Rayfield

Correspondence

andre.rowe@bristol.ac.uk

In brief

Rowe and Rayfield reveal two evolutionary paths to theropod dominance: tyrannosaurids crushed prey with high-stress skulls, whereas other clades sliced with low-stress bites. These biomechanical extremes underscore how feeding power shaped Mesozoic ecosystems.

Rowe & Rayfield, 2025, Current Biology *35*, 3664–3673
August 4, 2025 © 2025 The Author(s). Published by Elsevier Inc.
https://doi.org/10.1016/j.cub.2025.06.051

CellPress

CellPress
OPEN ACCESS

Current Biology

Article

Carnivorous dinosaur lineages adopt different skull performances at gigantic size

Andre J. Rowe[1,2,3,*] **and Emily J. Rayfield**[1]
[1]School of Earth Sciences, University of Bristol, Bristol BS8 1RJ, UK
[2]Senior author
[3]Lead contact
*Correspondence: andre.rowe@bristol.ac.uk
https://doi.org/10.1016/j.cub.2025.06.051

SUMMARY

Theropoda is one of the most extensively studied dinosaur clades, including iconic carnivores such as *Tyrannosaurus rex* and *Spinosaurus aegyptiacus*. The clade includes the largest terrestrial bipeds ever described, including three lineages that independently achieved giant size: Megalosauroidea, Allosauroidea, and Tyrannosauroidea. Here, we investigate how increasing size influenced feeding performance by quantifying feeding-induced mechanical performance across numerous large theropods using 3D finite element analysis. Unexpectedly, we discovered a divergence in functional strategy among the three lineages that led to gigantic top predators: in non-tyrannosauroid theropods, skull stress generally did not increase with size, in contrast to tyrannosauroids, which experienced greater stress due to increased muscle volume and bite forces. When skulls were scaled to equivalent size, smaller theropods, particularly basal taxa, experienced higher stresses. Despite similar scaling constraints, theropods adopted two distinct functional and likely ecological strategies: increased size with reduced stress or increased skull size, muscle volume, and bite force at the cost of higher stress. Giant tyrannosaurids uniquely maximized bite force despite elevated cranial stress, a strategy perhaps driven by the demands of subduing increasingly large and mobile prey in the Late Cretaceous. Alternatively—or additionally—this shift may reflect ecological displacement by coexisting predators such as smaller theropods and giant crocodyliforms. Whatever the cause, tyrannosaurids pursued a high-risk, high-reward feeding strategy unlike any seen in their Early Cretaceous counterparts, underscoring a profound shift in mega-carnivore evolution near the end of the Mesozoic.

INTRODUCTION

Theropod dinosaurs are among the most iconic fossil organisms, known for their repeated evolution of large body size and for producing the largest bipedal carnivores in Earth's history.[1–4] Unambiguous theropods appeared in the Late Triassic (~228 Ma),[5] and by the Early Jurassic, genera such as *Dilophosaurus* were the largest terrestrial predators.[6] Multiple theropod clades independently evolved giant forms by the Late Cretaceous, including the ~5,600-kg allosauroid *Acrocanthosaurus*[7,8] and the ~8,000-kg megalosauroid *Spinosaurus*.[9]

Body size influences many aspects of organismal biology, affecting predation, energetics, and extinction risk.[10–13] Although Cope's rule—size increase over time—is not universal across vertebrates, it appears to apply to several theropod lineages.[3,14] Nonetheless, small-bodied theropods persisted throughout the Mesozoic, reflecting persistent ecological differentiation and functional diversity among carnivorous dinosaurs.[15,16] Such coexistence likely involved partitioning of prey size, hunting strategies, or habitat use, reducing direct competition with their larger counterparts. At least three major theropod clades independently evolved large size: megalosauroids, allosauroids, and tyrannosauroids.[17,18] These shifts occurred across time and geography and involved significant changes in skull morphology. For example, basal tyrannosauroids were lightly built (~125 kg), whereas *Tyrannosaurus* reached ~9,500 kg with deep, massively muscled skulls.[19–22]

Although previous studies have examined how size affects locomotion,[23,24] feeding biomechanics at large size remain less understood. Bite force scales with body mass in extant diapsids,[25–28] but many prior theropod studies have relied on 2D shape approximations.[29–33] Modern 3D technologies—computed tomography (CT), surface scanning, photogrammetry—now allow detailed analyses of skull mechanics using finite element analysis (FEA) across a broad taxonomic sample.[34–42] Yet, fundamental questions remain: how does skull performance scale with body size? Do large theropods reduce stress via geometric scaling or do muscle forces increase proportionally, leading to higher stress loads? Does cranial morphology evolve to offset these forces?

Here, we used FEA to test how body size relates to cranial biomechanics across 18 theropods spanning key clades that independently evolved large size (Megalosauridae, Allosauroidea, and Tyrannosauroidea), along with smaller basal taxa such as *Herrerasaurus* and *Dilophosaurus* for comparison (Figure 1). We excluded likely herbivorous theropods like therizinosaurs, which possessed specialized tooth and muscle arrangements adapted for herbivory.[43,44]

 © 2025 The Author(s). Published by Elsevier Inc.
This is an open access article under the CC BY license (http://creativecommons.org/licenses/by/4.0/).

CellPress
OPEN ACCESS

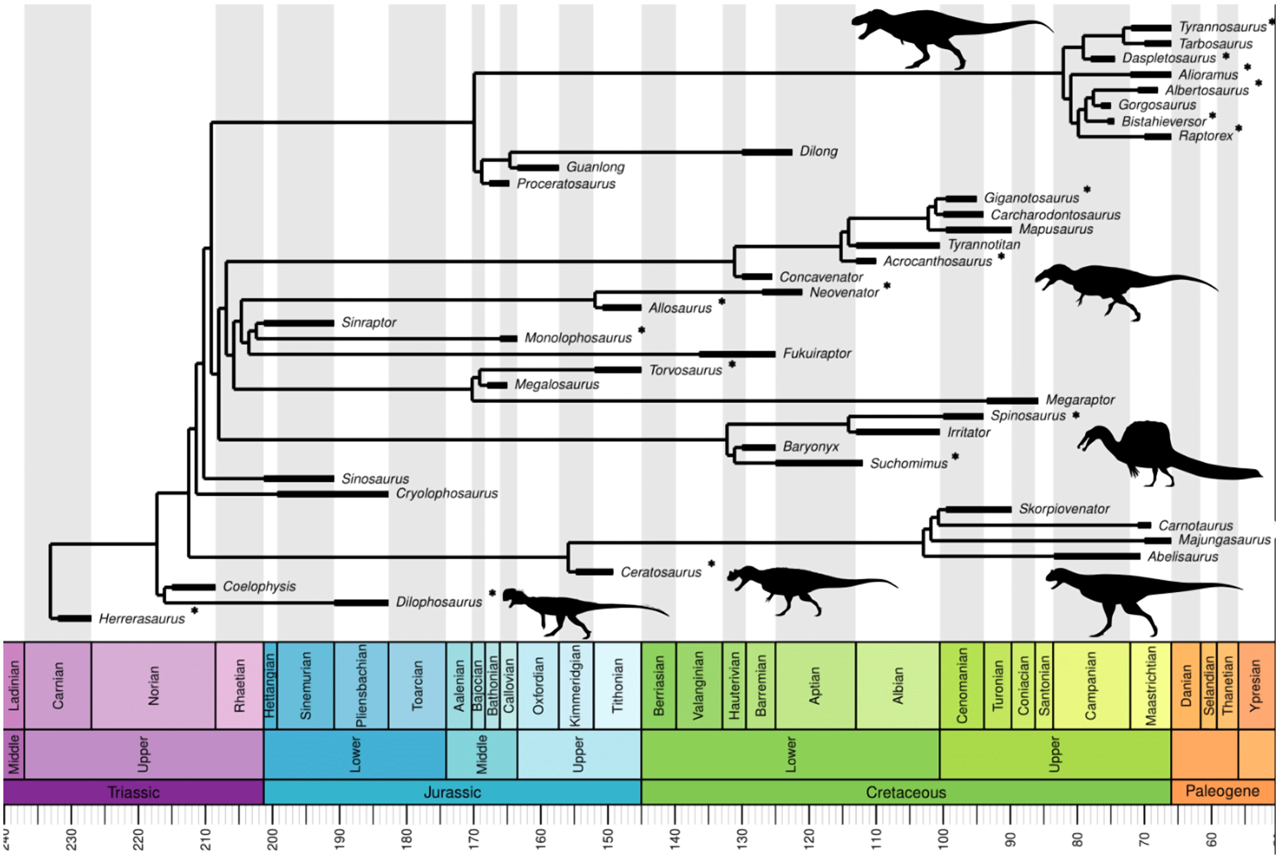

Figure 1. Temporally calibrated theropod dinosaur phylogeny

Phylogeny based on several recent studies (Brusatte and Carr,[45] Hendrickx et al.,[46] and Tanaka et al.[47]). Asterisks indicate taxa included in this study. Theropod silhouettes from PhyloPic.

We test three key hypotheses using FEA-based 3D mechanical models: (1) stress reduction with size—larger taxa should experience lower stress under geometric scaling predictions; (2) elevated stress in tyrannosauroids—if bite forces scale disproportionately in tyrannosaurids, they should show higher cranial stress relative to similarly sized taxa; and (3) higher stress in smaller taxa when size corrected—when skulls are scaled to the same size, smaller taxa should show higher stress due to less robust skull geometry.

RESULTS

Crania at actual size

Von Mises stress patterns were broadly consistent across taxa (Figure 2), with *Herrerasaurus* displaying the highest cranial stresses, particularly at the rear of the cranium. Large tetanurans such as *Acrocanthosaurus* and *Suchomimus* experienced relatively low cranial stress overall. High-stress areas were concentrated around the quadrate and jugal bones, especially in large tyrannosaurids and allosauroids (*Allosaurus* and *Neovenator*).

Mean maximum principal strain was calculated for the crania and mandibles (Figure 3). Mean maximum principal strain was highest in *Herrerasaurus*, with *Monolophosaurus*, *Ceratosaurus*, and *Spinosaurus* also showing elevated strain. Larger theropods (*Suchomimus*, *Acrocanthosaurus*, and *Giganotosaurus*) generally experienced lower strain, but no clear size-related trend was observed.

Calculating a linear regression with all skull lengths (x) and cranial von Mises stresses (y) yielded a p value of 0.3305, whereas the p value when tyrannosauroids were excluded yielded a p value of 0.2596, both indicating a weak relationship between the two. A linear regression analysis of the crania reveals a coefficient of determination value of $R^2 = 0.0581$. Phylogenetically corrected regression analysis incorporating skull lengths and cranial von Mises stresses gave a p value of 0.5227, confirming low significance.

Mandibles at actual size

Mandibles exhibited higher stresses than crania, with stress concentrated near the mandibular fenestrae, especially in *Dilophosaurus* and *Suchomimus*—likely due to the presence of wrapping pterygoid muscles. In allosauroids (e.g., *Acrocanthosaurus*), stress was concentrated toward the anterior jaw, whereas in tyrannosauroids (e.g., *Daspletosaurus*), stress was more pronounced at the posterior end.

As in previous plots, *Herrerasaurus* experiences the highest values, with certain medium-sized theropods also exhibiting high mandibular strain, including *Neovenator*. *Bistahieversor*

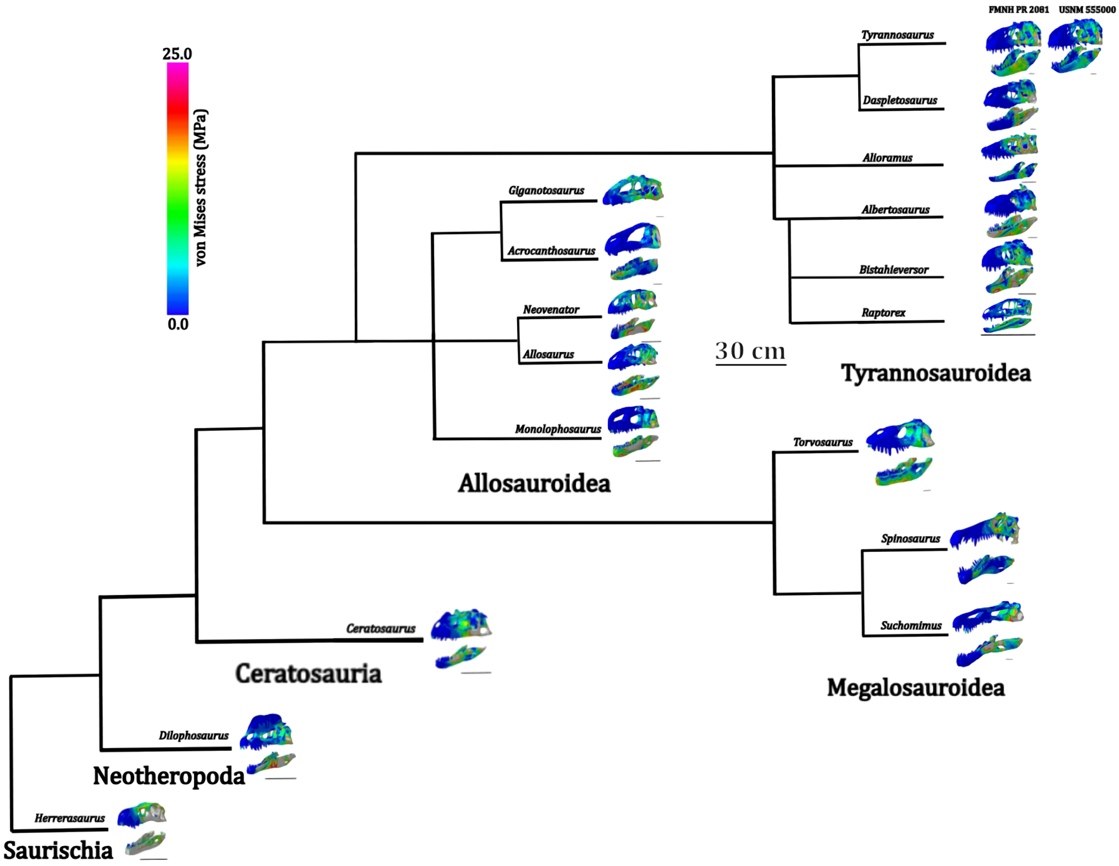

Figure 2. Finite element stress maps for all theropod skulls modeled at actual size after simulated adductor contraction and biting at the two front teeth

Areas of high stress are indicated by hotter colors, and low stress is indicated by cooler colors. Gray regions indicate regions with the highest probabilities of structural failure. Note the very high stresses occurring in the basal ambiguous theropod *Herrerasaurus*.
For larger stress maps of actual-size skulls, see Figure S1.

demonstrated the highest mandibular strain of the large-bodied theropods.

Calculating a linear regression of non-tyrannosauroid theropods with skull lengths (x) and mandibular von Mises stresses (y) yielded a p value of 0.0163, indicating a highly statistically significant relationship between skull length and mandible stress. When tyrannosauroids were included in the calculation, a p value of 0.0628 was obtained, weakening the relationship. Phylogenetically corrected regression analysis gave a p value of 0.184, indicating weak significance.

Crania when scaled to equivalent size

These models assess how theropod skull shapes respond to feeding loads when skulls are scaled to the same equivalent size (equal muscle force to surface area ratio) (Figures 4 and S1).

Herrerasaurus and *Ceratosaurus*, two of the shortest skulls, demonstrate the highest stress values, whereas the large megalosauroids and allosauroids are generally the most stress resistant, especially *Acrocanthosaurus*. Tyrannosauroids generally become more stressed at smaller body size, such as *Raptorex* and *Alioramus*; *Tyrannosaurus* specimens exhibited low-stress levels, comparable to large megalosauroids and allosauroids. High stress remained concentrated near the quadrate and jugal bones, like the unscaled results.

Cranial strain results for the muscle-force-scaled dataset revealed the highest strain in *Herrerasaurus*, *Ceratosaurus*, and *Neovenator* (Figures 5 and S2). Lowest strains were present in *Acrocanthosaurus* and *Suchomimus*. Strain results for the muscle-force-scaled mandibles yielded the highest strains in *Herrerasaurus* and *Neovenator*. The lowest values were reported in *Spinosaurus* and *Acrocanthosaurus*, two of the largest taxa in the dataset.

Calculating a linear regression with skull lengths (x) and cranial von Mises stresses (y) for the scaled muscle force dataset yielded a p value of 0.1981, whereas the p value when tyrannosauroids were excluded resulted in 0.2976, both indicating weak significance. Phylogenetically corrected regression analysis gives us a p value of 0.4376, confirming low statistical significance.

Mandibles when scaled to equivalent size

Scaling to the muscle force to surface area ratio of *Tyrannosaurus* resulted in structural failure in the mandibles of smaller- and medium-sized taxa, including *Monolophosaurus*, *Neovenator*, *Allosaurus*, *Ceratosaurus*, and *Herrerasaurus*. Smaller

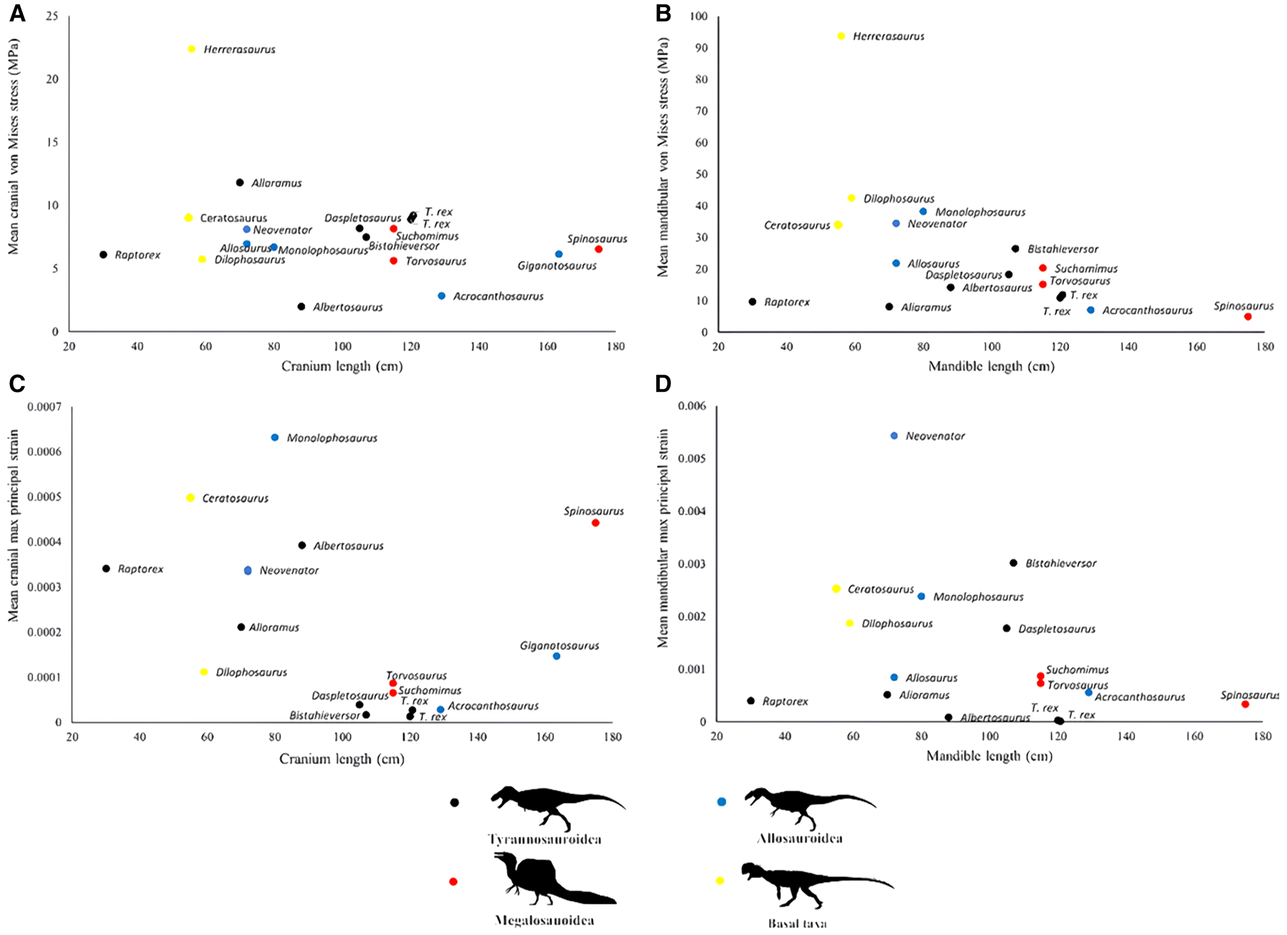

Figure 3. Theropod skull lengths versus von Mises stresses at actual size

Cranial lengths plotted against mesh-weighted mean cranial von Mises stresses (A), mean mandible von Mises stress (B), mean cranial maximum principal strain (C), and mean mandible principal strain for each theropod dinosaur at actual size (D). *Herrerasaurus* is not included in (C) and (D) due to its high values. Skull lengths in cm. Note that strain is a unitless quantity. Theropod silhouettes from PhyloPic by Scott Hartman.

tyrannosaurids (*Raptorex* and *Alioramus*) also experienced high stress. Larger non-tyrannosauroid tetanurans (*Spinosaurus* and *Acrocanthosaurus*) showed more even stress distribution along the mandible. *Dilophosaurus* exhibited high stress at the posterior, decreasing toward the anterior.

Consistent with other datasets, *Herrerasaurus* exhibited the highest mandibular strain, followed by *Neovenator*. Among large theropods, *Bistahieversor* demonstrated the highest mandibular strain, potentially due to 3D model reconstruction. *Dilophosaurus* also showed elevated strain.

Calculating a linear regression with skull lengths (x) and mandibular von Mises stresses (y) for the scaled muscle force dataset yielded a p value of 0.2411 and, when tyrannosauroids were excluded, a p value of 0.3046, both indicating weak significance. Phylogenetically corrected regression analysis gives a p value of 0.494, confirming low significance.

DISCUSSION

Non-avian theropod dinosaurs represent a classic example of Cope's rule, exhibiting a macroevolutionary trend toward increasing body size through time. This study presents the largest 3D dataset of carnivorous theropod skulls analyzed using finite element analysis (FEA) to explore cranial and mandibular stress under feeding-related loads. Our findings are the first to document biomechanical performance across a broad phylogenetic and size range of theropods, revealing distinct functional patterns associated with body size evolution.

The greatest cranial stress resistance is observed in large-bodied non-tyrannosauroid theropods, particularly megalosauroids and allosauroids such as *Spinosaurus* and *Acrocanthosaurus*. These taxa exhibit relatively low von Mises stress during simulated feeding, especially in the cranium. In contrast, large tyrannosauroids display elevated cranial stress levels, largely attributable to their expanded posterior skull regions, which accommodate more adductor musculature and generate correspondingly higher bite forces. Despite this, stress magnitudes in all taxa remain within biomechanically tolerable limits.

These results reveal two divergent functional strategies among large-bodied theropods: low stress, lower-power strategy—typified by allosauroids, megalosauroids, and basal taxa,

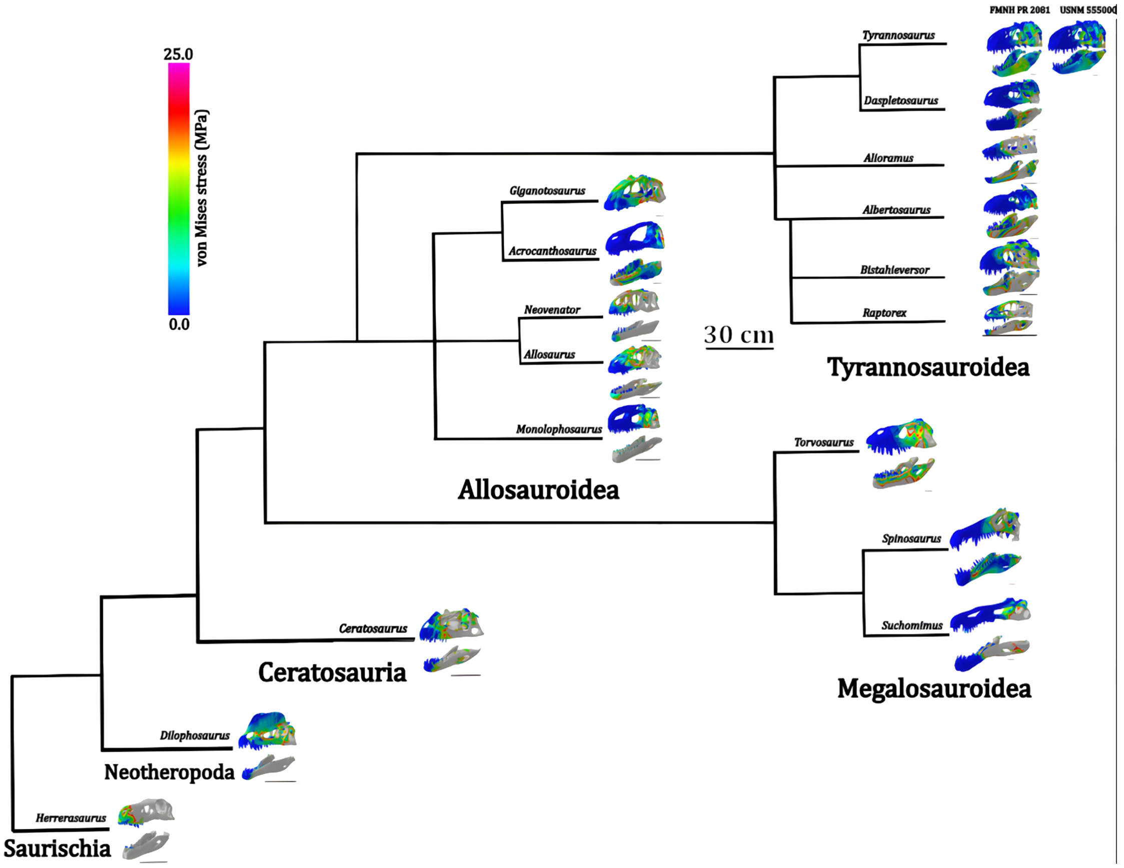

Figure 4. Finite element stress maps for all theropod skulls modeled when size corrected

Gray regions indicate regions with the highest probabilities of structural failure. For larger stress maps of size-corrected skulls, see Figure S2.

whose slenderer skulls and more distributed muscle loading reduce peak stresses; high-stress, high-power strategy—represented by tyrannosaurids, whose posteriorly expanded crania allow for greater adductor muscle mass and stronger, bone-crushing bites.

Our hypotheses are partially supported: the null hypothesis (1) is partially falsified, whereas hypotheses (2) and (3) are upheld. Large non-tyrannosauroids indeed exhibit lower stress magnitudes compared with similarly sized tyrannosauroids, and smaller theropods, once size-corrected, tend to approach stress thresholds that might increase failure risk.

Functional divergence likely reflects ecological specialization. Tyrannosaurids, including *Tyrannosaurus rex*, combined stiff, akinetic skulls with high bite forces (up to ∼60,000 N) facilitated by fused nasals and widely spaced quadrates, allowing efficient force transmission during predation.[48–50] These features point toward possible ambush predation strategies, consistent with low rotational inertia, limited cursorial ability, and fossil evidence of active predation.[24,51,52] Subadult and slender-skulled forms such as alioramins may have pursued more agile prey.[53,54] When scaled to actual size, tyrannosauroids continue to show high biting stress, yet their cranial architecture efficiently manages these loads. Their distinctive traits—fused cranial elements, robust mandibles, and posteriorly expanded skulls—distinguish them biomechanically from other large theropods. In contrast, large tetanurans like *Allosaurus* and *Monolophosaurus* likely adopted more generalized feeding strategies involving lower bite forces and slashing bites, aided by wider gapes, cranial flexibility, and avian-like prey manipulation.[55,56] The elevated skull stress observed in *Herrerasaurus* is notable given its phylogenetic position. As a basal taxon, it retains plesiomorphic features that may predispose its skull to higher mechanical stress under simulated biting loads. Although model reconstructions were conservative and guided by phylogenetic bracketing, some exaggeration of stress magnitudes is possible due to those reconstructions, particularly in regions influencing stress distribution.[35]

Mandibular stress trends differ between lineages. Although tyrannosauroid mandibles show increasing stress with body size, large tetanuran mandibles show a statistically significant trend toward reduced stress. This may reflect differences in prey acquisition tactics: stiff, puncture-pull biting in tyrannosaurids versus slashing or tearing in allosauroids, where broader gape mechanics and kinetic cranial elements may have modulated stress distributions.

Medium-sized theropods (e.g., small allosauroids) experience skull stresses comparable to their larger relatives, implying overlapping feeding strategies or targeting of similar prey resources. The apparent ecological success of tyrannosauroids and

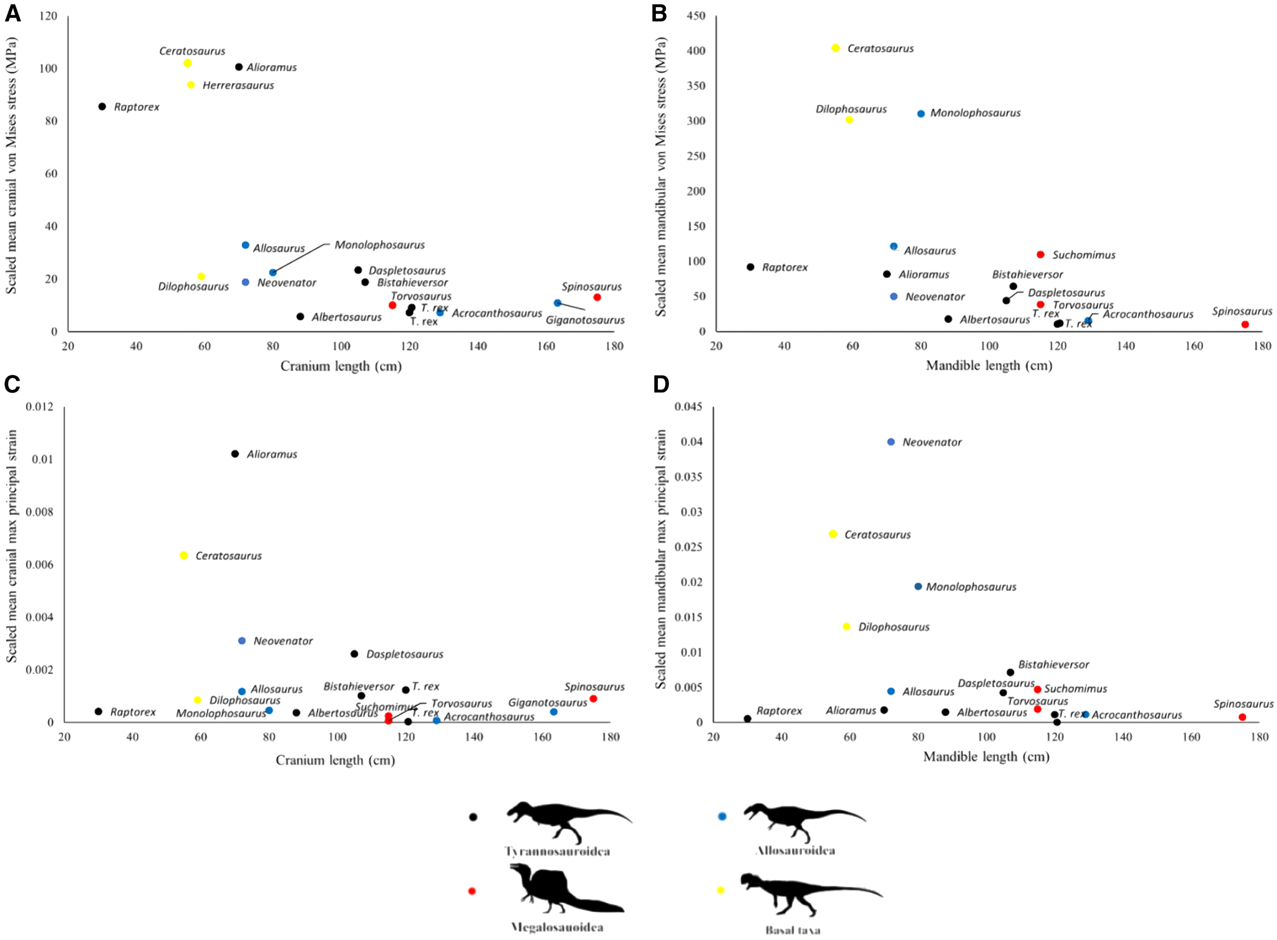

Figure 5. Theropod skull lengths versus von Mises stresses when size corrected

Skull lengths plotted against calculated mesh-weighted mean cranial von Mises stress (A), mean mandibular von Mises stress (B), mean cranial maximum principal strain (C), and mean mandibular maximum principal strain for each theropod dinosaur cranium when size corrected (D). *Herrerasaurus* is not included in (B), (C), and (D) due to its high values. Skull lengths in cm. Theropod silhouettes from PhyloPic by Scott Hartman.

megalosauroids may partly reflect biomechanical performance advantages at large body sizes, potentially enabling them to displace or outcompete mid-sized clades such as abelisauroids—a pattern reflected in the relative scarcity of mid-sized carnivores in some ecosystems.[57] Spinosaurids, in contrast, are often interpreted as semi-aquatic piscivores.[58–62] Their elongated rostra and reduced antorbital fenestrae contribute to increased torsional resistance despite low bite forces relative to other large theropods.[63] Although often associated with aquatic prey, fossil evidence, e.g., gut contents containing pterosaurs and iguanodontids, suggests a broader dietary repertoire.[64–66] Their cranial morphology thus reflects both specialization and flexibility.

Overall, large body size conferred biomechanical advantages across theropod lineages by enhancing muscle mass and bite force, enabling broader prey spectra. However, skull shape also constrained performance. Taxa with short, slender skulls underperformed when size-corrected, indicating shape-function interactions. Similar conclusions were drawn by Ma et al.,[31] who identified a trend of jaw strengthening across theropod clades.

Morphologically, phylogeny appears to drive skull form more than function at broad scales,[67] but our results suggest a tighter link between function, form, and ecology at finer phylogenetic resolutions. Similarly, Lautenschlager[68] found that orbit shape, e.g., elliptical or keyhole shaped, enhances stress dissipation, particularly in large-bodied taxa—reinforcing the idea that skull design evolved under complex, size-dependent constraints. Mandibular and cranial elements reveal differing biomechanical patterns, likely due to their distinct functional roles.

The vertebrate mandible is assumed to be more closely adapted for feeding function, whereas the cranium is subject to multiple constraints[69]; hence, our cranial and mandibular results do not demonstrate identical trends. The mandible, primarily adapted for feeding, shows a clearer relationship with body size and stress. In contrast, the cranium—subject to multiple selective pressures, including sensory and display functions—shows weaker size-stress correlations. These findings support the view that cranial morphology in theropods evolved under multifunctional constraints.

Cranial ornamentation, such as crests and horns (e.g., in *Dilophosaurus* and *Ceratosaurus*), appears to have limited

Table 1. Skull lengths and body mass estimates for each taxon ordered by phylogenetic position

Specimen name	Specimen number	Skull length (cm)	Body mass estimate (kg)	Body mass source
Herrerasaurus ischigualastensis	PVSJ 407	56	350	Paul[76]
Dilophosaurus wetherilli	UCMP 77270	59	400	Paul[76]
Ceratosaurus nasicornis	BYUVP 12893	55	418	Therrien and Henderson[8]
Monolophosaurus jiangi	IVPP 84019	80	475	Paul[76]
Allosaurus jimmadseni	MOR 693	72	1,500	Bates et al.[77]
Neovenator salerii	MIWG.6348	72	1,000	Paul[76]
Acrocanthosaurus atokensis	NCSM 14345	129	2,551.25	Snively et al.[51]
Giganotosaurus carolinii	MUCPv-Ch1	163.4	3,540.64	Snively et al.[51]
Torvosaurus gurneyi	BYUVP 2002–2014, 2016–2018, 4882–4884, 4860, 4890, 4998, 5110, 5286, 9120–9122, 9249	115	1,650	Hendrickx and Mateus[78]
Spinosaurus aegyptiacus	FSAC-KK 11888	175	7,400	Sereno et al.[62]
Suchomimus tenerensis	MNN GDF501	119	3,816.1	Seebacher[79]
Raptorex kriegsteini	LH PV18	30	65	Sereno et al.[80]
Bistahieversor sealeyi	NMMNH P-27469	107	3,300	Molina-Pérez et al.[81]
Albertosaurus sarcophagus	TMP 1981.010.0001	88	1,685	Christiansen and Fariña[7]
Alioramus altai	IGM 100/1844	70	369	Brusatte et al.[82]
Daspletosaurus torosus	FMNH PR308	105	2,700	Christiansen and Fariña[7]
Tyrannosaurus rex	USNM 555000	120	6,216	Campione et al.[83]
Tyrannosaurus rex	FMNH PR 2081	127.5	7,377	Campione et al.[83]

Body masses were referenced from the literature and skull lengths were measured in MeshLab 2020.06.

impact on feeding performance. Stress values in ornamented taxa are comparable to those of similarly sized, unornamented theropods. Although Johnson-Ransom et al.[41] suggested a potential stress-reducing role for the crest in *Proceratosaurus*, this may represent a lineage-specific adaptation rather than a general functional pattern.

Conclusion

Large theropods evolved divergent biomechanical strategies as they attained gigantism, reflecting distinct ecological and evolutionary pressures. Tyrannosaurids, in particular, developed deep, powerfully muscled skulls that tolerated high levels of feeding-induced stress—an adaptation consistent with bone-crunching predation and the exploitation of giant prey. This shift toward maximizing bite force may have been driven by the unique demands of Late Cretaceous ecosystems, including competition with smaller theropods and giant crocodyliforms, as well as the emergence of heavily armored herbivores like ankylosaurs. In contrast, other giant theropods—such as allosauroids and megalosauroids—retained more generalized cranial architectures that experienced lower stress, suggesting functional strategies optimized for less-force-intensive feeding. Although cranial biomechanics was not the only factor shaping body size evolution in Theropoda, our results indicate that feeding performance played a key role in determining how different lineages achieved—and sustained—ecological dominance. These contrasting strategies highlight multiple evolutionary pathways to mega-carnivory and underscore the deep functional diversity that underpinned the success of large-bodied predatory dinosaurs.

RESOURCE AVAILABILITY

Lead contact

Further information and requests for resources should be directed to and will be fulfilled by the lead contact, Andre J. Rowe (andre.rowe@bristol.ac.uk).

Materials availability

This study did not generate new reagents.

Data and code availability

- Dinosaur skull STL files are available with permission from their respective institutions where scanning took place.
- No original code was generated in this study.
- Any additional information required to reanalyze the data reported in this paper is available from the lead contact upon request.

ACKNOWLEDGMENTS

We thank Matteo Fabbri for the *Spinosaurus* skull data, Martin Hunt for the *Neovenator* skull data, Stephan Lautenschlager for the *Allosaurus* skull data, and Nick Wiersum for access and assistance with scanning various specimens at the Dinosaur Discovery Museum in Kenosha, Wisconsin. Thanks to Eric Snively for both the *Giganotosaurus* cranium and his always helpful advice. Thanks to Alex Hastings for access to the *Dilophosaurus* skull replica at the Science Museum of Minnesota, Patricia Burke for access to the *Monolophosaurus* replica at the Milwaukee Public Museum, and William Simpson for access to specimens at the Field Museum of Natural History, which allowed us to scan the *Daspletosaurus* replica skull. We also thank Pete Makovicky for sending us CT models of *Tyrannosaurus* FMNH PR 2081 and Holly Little for sending us *Tyrannosaurus* USNM 555000 models. Thanks to Carolyn Merrill and Mark Norell for access to the *Alioramus* CT data, Bernard K. Means for uploading and sending the *Albertosaurus* skull model, Thomas Williamson for all *Bistahieversor* CT data, including STL files and the original TIFF files, and to Elizabeth Martin-Silverstone for receiving and storing that data. Thank you to

CellPress
OPEN ACCESS

Mike Benton for his helpful advice during the submission process of the cover letter and manuscript, as well as his mentorship during A.J.R.'s PhD at the University of Bristol. Lastly, we thank our two anonymous reviewers whose comments improved the clarity of this manuscript. E.J.R. was funded by the Biotechnology and Biological Sciences Research Council (grant no. BB/W00867X/1).

AUTHOR CONTRIBUTIONS

A.J.R. and E.J.R. conceived the research. E.J.R. supervised the research. A.J.R. performed the research. A.J.R. and E.J.R. analyzed the data. A.J.R. wrote the paper.

DECLARATION OF INTERESTS

The authors declare no competing interests.

STAR★METHODS

Detailed methods are provided in the online version of this paper and include the following:

- KEY RESOURCES TABLE
- EXPERIMENTAL MODEL AND STUDY PARTICIPANT DETAILS
  - Taxonomic Sampling
- METHOD DETAILS
  - 3D imaging and model generation
  - Model processing and editing
  - Finite element analysis (FEA)
  - Limitations of the Study
  - Data analysis
- QUANTIFICATION AND STATISTICAL ANALYSIS
- ADDITIONAL RESOURCES

SUPPLEMENTAL INFORMATION

Supplemental information can be found online at https://doi.org/10.1016/j.cub.2025.06.051.

Received: May 12, 2025
Revised: June 9, 2025
Accepted: June 19, 2025
Published: August 4, 2025

REFERENCES

1. Sereno, P.C. (1999). The evolution of dinosaurs. Science *284*, 2137–2147. https://doi.org/10.1126/science.284.5423.2137.
2. Benson, R.B.J., Campione, N.E., Carrano, M.T., Mannion, P.D., Sullivan, C., Upchurch, P., and Evans, D.C. (2014a). Rates of dinosaur body mass evolution indicate 170 million years of sustained ecological innovation on the avian stem lineage. PLoS Biol. *12*, e1001853. https://doi.org/10.1371/journal.pbio.1001853.
3. Benson, R.B.J., Hunt, G., Carrano, M.T., and Campione, N. (2018). Cope's rule and the adaptive landscape of dinosaur body size evolution. Palaeontology *61*, 13–48. https://doi.org/10.1111/pala.12329.
4. Cullen, T.M., Canale, J.I., Apesteguía, S., Smith, N.D., Hu, D., and Makovicky, P.J. (2020). Osteohistological analyses reveal diverse strategies of theropod dinosaur body-size evolution. Proc. Biol. Sci. *287*, 20202258. https://doi.org/10.1098/rspb.2020.2258.
5. Martínez, R.N., Sereno, P.C., Alcober, O.A., Colombi, C.E., Renne, P.R., Montañez, I.P., and Currie, B.S. (2011). A basal dinosaur from the dawn of the dinosaur era in Southwestern Pangaea. Science *331*, 206–210. https://doi.org/10.1126/science.1198467.
6. Marsh, A.D., and Rowe, T.B. (2020). A comprehensive anatomical and phylogenetic evaluation of *Dilophosaurus wetherilli* (Dinosauria, Theropoda) with descriptions of new specimens from the Kayenta Formation of northern Arizona. J. Paleontol. *94*, 1–103. https://doi.org/10.1017/jpa.2020.14.
7. Christiansen, P., and Fariña, R.A. (2004). Mass prediction in theropod dinosaurs. Hist. Biol. *16*, 85–92. https://doi.org/10.1080/08912960412331284313.
8. Therrien, F., and Henderson, D.M. (2007). My theropod is bigger than yours... or not: Estimating body size from skull length in theropods. J. Vertebr. Paleontol. *27*, 108–115. https://doi.org/10.1671/0272-4634(2007)27[108:MTIBTY]2.0.CO;2.
9. Dal Sasso, C.D., Maganuco, S., Buffetaut, E., and Mendez, M.A. (2005). New information on the skull of the enigmatic theropod *Spinosaurus*, with remarks on its size and affinities. J. Vertebr. Paleontol. *25*, 888–896. https://doi.org/10.1671/0272-4634(2005)025[0888:NIOTSO]2.0.CO;2.
10. Peters, R.H. (1986). The Ecological Implications of Body Size (Cambridge University Press).
11. Brown, J.H., and Sibly, R.M. (2006). Life-history evolution under a production constraint. Proc. Natl. Acad. Sci. USA *103*, 17595–17599. https://doi.org/10.1073/pnas.0608522103.
12. Ripple, W.J., Wolf, C., Newsome, T.M., Hoffmann, M., Wirsing, A.J., and McCauley, D.J. (2017). Extinction risk is most acute for the world's largest and smallest vertebrates. Proc. Natl. Acad. Sci. USA *114*, 10678–10683. https://doi.org/10.1073/pnas.1702078114.
13. Case, T.J. (1979). Optimal body size and an animal's diet. Acta Biotheor. *28*, 54–69. https://doi.org/10.1007/BF00054680.
14. Hone, D.W.E., and Benton, M.J. (2005). The evolution of large size: how does Cope's Rule work? Trends Ecol. Evol. *20*, 4–6. https://doi.org/10.1016/j.tree.2004.10.012.
15. Brusatte, S.L., Carr, T.D., Erickson, G.M., Bever, G.S., and Norell, M.A. (2009). A long-snouted, multihorned tyrannosaurid from the Late Cretaceous of Mongolia. Proc. Natl. Acad. Sci. USA *106*, 17261–17266. https://doi.org/10.1073/pnas.0906911106.
16. Hone, D.W.E., Keesey, T.M., Pisani, D., and Purvis, A. (2005). Macroevolutionary trends in the Dinosauria: Cope's rule. J. Evol. Biol. *18*, 587–595. https://doi.org/10.1111/j.1420-9101.2004.00870.x.
17. Erickson, G.M., Makovicky, P.J., Currie, P.J., Norell, M.A., Yerby, S.A., and Brochu, C.A. (2004). Gigantism and comparative life-history parameters of tyrannosaurid dinosaurs. Nature *430*, 772–775. https://doi.org/10.1038/nature02699.
18. D'Emic, M.D., O'Connor, P.M., Sombathy, R.S., Cerda, I., Pascucci, T.R., Varricchio, D., Pol, D., Dave, A., Coria, R.A., and Curry Rogers, K.A. (2023). Developmental strategies underlying gigantism and miniaturization in non-avialan theropod dinosaurs. Science *379*, 811–814. https://doi.org/10.1126/science.adc8714.
19. Persons, W.S., IV, Currie, P.J., and Erickson, G.M. (2020). An older and exceptionally large adult specimen of *Tyrannosaurus rex*. Anat. Rec. (Hoboken) *303*, 656–672. https://doi.org/10.1002/ar.24118.
20. Xu, X., Norell, M.A., Kuang, X., Wang, X., Zhao, Q., and Jia, C. (2004). Basal tyrannosauroids from China and evidence for protofeathers in tyrannosauroids. Nature *431*, 680–684. https://doi.org/10.1038/nature02855.
21. Xu, X., Clark, J.M., Forster, C.A., Norell, M.A., Erickson, G.M., Eberth, D.A., Jia, C., and Zhao, Q. (2006). A basal tyrannosauroid dinosaur from the Late Jurassic of China. Nature *439*, 715–718. https://doi.org/10.1038/nature04511.
22. Paul, G.S. (2016). The Princeton Field Guide to Dinosaurs (Princeton University Press), pp. 104–105.
23. Alexander, R.M. (1991). How dinosaurs ran. Sci. Am. *264*, 130–136. https://doi.org/10.1038/scientificamerican0491-130.
24. Hutchinson, J.R., and Garcia, M. (2002). *Tyrannosaurus* was not a fast runner. Nature *415*, 1018–1021. https://doi.org/10.1038/4151018a.
25. Erickson, G.M., Lappin, A.K., and Vliet, K.A. (2003). The ontogeny of bite-force performance in American alligator (*Alligator mississippiensis*). J. Zool. *260*, 317–327. https://doi.org/10.1017/S0952836903003819.
26. Gignac, P.M., and Erickson, G.M. (2016). Ontogenetic bite-force modeling of *Alligator mississippiensis*: implications for dietary transitions in a

large-bodied vertebrate and the evolution of crocodylian feeding. J. Zool. *299*, 229–238. https://doi.org/10.1111/jzo.12349.

27. Deeming, D.C. (2022). Inter-relationships among body mass, body dimensions, jaw musculature and bite force in reptiles. J. Zool. *318*, 23–33. https://doi.org/10.1111/jzo.12981.
28. Sakamoto, M. (2022). Estimating bite force in extinct dinosaurs using phylogenetically predicted physiological cross-sectional areas of jaw adductor muscles. PeerJ *10*, e13731. https://doi.org/10.7717/peerj.13731.
29. Rayfield, E.J. (2005). Aspects of comparative cranial mechanics in the theropod dinosaurs *Coelophysis*, *Allosaurus* and *Tyrannosaurus*. Zool. J. Linn. Soc. *144*, 309–316. https://doi.org/10.1111/j.1096-3642.2005.00176.x.
30. Rayfield, E.J. (2011). Structural performance of tetanuran theropod skulls, with emphasis on the Megalosauridae, Spinosauridae and Carcharodontosauridae. In Studies on fossil Tetrapods, *86*, P.M. Barrett, and A.R. Milner, eds. (Special Papers in Palaeontology), pp. 241–253.
31. Ma, W., Pittman, M., Butler, R.J., and Lautenschlager, S. (2022). Macroevolutionary trends in theropod dinosaur feeding mechanics. Curr. Biol. *32*, 677–686.e3. https://doi.org/10.1016/j.cub.2021.11.060.
32. Rowe, A.J., and Snively, E. (2022). Biomechanics of juvenile tyrannosaurid mandibles and their implications for bite force: Evolutionary biology. Anat. Rec. (Hoboken) *305*, 373–392. https://doi.org/10.1002/ar.24602.
33. Tse, Y.T., Miller, C.V., and Pittman, M. (2024). Morphological disparity and structural performance of the dromaeosaurid skull informs ecology and evolutionary history. BMC Ecol. Evol. *24*, 39. https://doi.org/10.1186/s12862-024-02222-5.
34. Cunningham, J.A., Rahman, I.A., Lautenschlager, S., Rayfield, E.J., and Donoghue, P.C.J. (2014). A virtual world of paleontology. Trends Ecol. Evol. *29*, 347–357. https://doi.org/10.1016/j.tree.2014.04.004.
35. Rowe, A.J., and Rayfield, E.J. (2022). The efficacy of computed tomography scanning versus surface scanning in 3D finite element analysis. PeerJ *10*, e13760. https://doi.org/10.7717/peerj.13760.
36. Ross, C.F. (2005). Finite element analysis in vertebrate biomechanics. Anat. Rec. A Discov. Mol. Cell. Evol. Biol. *283*, 253–258. https://doi.org/10.1002/ar.a.20177.
37. Rayfield, E.J. (2007). Finite element analysis and understanding the biomechanics and evolution of living and fossil organisms. Annu. Rev. Earth Planet. Sci. *35*, 541–576. https://doi.org/10.1146/annurev.earth.35.031306.140104.
38. Lautenschlager, S. (2013). Cranial myology and bite force performance of *Erlikosaurus andrewsi*: a novel approach for digital muscle reconstructions. J. Anat. *222*, 260–272. https://doi.org/10.1111/joa.12000.
39. Button, D.J., Barrett, P.M., and Rayfield, E.J. (2016). Comparative cranial myology and biomechanics of *Plateosaurus* and *Camarasaurus* and evolution of the sauropod feeding apparatus. Palaeontology *59*, 887–913. https://doi.org/10.1111/pala.12266.
40. Jamison-Todd, S., Moon, B.C., Rowe, A.J., Williams, M., and Benton, M.J. (2022). Dietary niche partitioning in Early Jurassic ichthyosaurs from Strawberry Bank. J. Anat. *241*, 1409–1423. https://doi.org/10.1111/joa.13744.
41. Johnson-Ransom, E., Li, F., Xu, X., Ramos, R., Midzuk, A.J., Thon, U., Atkins-Weltman, K., and Snively, E. (2024). Comparative cranial biomechanics reveal that Late Cretaceous tyrannosaurids exerted relatively greater bite force than in early-diverging tyrannosauroids. Anat. Rec. (Hoboken) *307*, 1897–1917. https://doi.org/10.1002/ar.25326.
42. Meade, L.E., Pittman, M., Balanoff, A., and Lautenschlager, S. (2024). Cranial functional specialisation for strength precedes morphological evolution in Oviraptorosauria. Commun. Biol. *7*, 436. https://doi.org/10.1038/s42003-024-06137-1.
43. Zanno, L.E., Gillette, D.D., Albright, L.B., and Titus, A.L. (2009). A new North American therizinosaurid and the role of herbivory in 'predatory' dinosaur evolution. Proc. Biol. Sci. *276*, 3505–3511. https://doi.org/10.1098/rspb.2009.1029.
44. Lautenschlager, S., Witmer, L.M., Altangerel, P., Zanno, L.E., and Rayfield, E.J. (2014). Cranial anatomy of *Erlikosaurus andrewsi* (Dinosauria, Therizinosauria): New insights based on digital reconstruction. J. Vertebr. Paleontol. *34*, 1263–1291. https://doi.org/10.1080/02724634.2014.874529.
45. Brusatte, S.L., and Carr, T.D. (2016). The phylogeny and evolutionary history of tyrannosauroid dinosaurs. Sci. Rep. *6*, 20252. https://doi.org/10.1038/srep20252.
46. Hendrickx, C., Mateus, O., and Buffetaut, E. (2016). Morphofunctional analysis of the quadrate of Spinosauridae (Dinosauria: Theropoda) and the presence of *Spinosaurus* and a second Spinosaurine taxon in the Cenomanian of North Africa. PLoS One *11*, e0144695. https://doi.org/10.1371/journal.pone.0144695.
47. Tanaka, K., Anvarov, O.U.O., Zelenitsky, D.K., Ahmedshaev, A.S., and Kobayashi, Y. (2021). A new carcharodontosaurian theropod dinosaur occupies apex predator niche in the early Late Cretaceous of Uzbekistan. R. Soc. Open Sci. *8*, 210923. https://doi.org/10.1098/rsos.210923.
48. Gignac, P.M., and Erickson, G.M. (2017). The biomechanics behind extreme osteophagy in *Tyrannosaurus rex*. Sci. Rep. *7*, 2012. https://doi.org/10.1038/s41598-017-02161-w.
49. Cost, I.N., Middleton, K.M., Sellers, K.C., Echols, M.S., Witmer, L.M., Davis, J.L., and Holliday, C.M. (2020). Palatal biomechanics and its significance for cranial kinesis in *Tyrannosaurus rex*. Anat. Rec. (Hoboken) *303*, 999–1017. https://doi.org/10.1002/ar.24219.
50. Snively, E., Henderson, D.M., and Phillips, D.S. (2006). Fused and vaulted nasals of tyrannosaurid dinosaurs: Implications for cranial strength and feeding mechanics. Palaeontol. Electron. *51*, 435–454.
51. Snively, E., O'Brien, H., Henderson, D.M., Mallison, H., Surring, L.A., Burns, M.E., Holtz, T.R., Russell, A.P., Witmer, L.M., Currie, P.J., et al. (2019). Lower rotational inertia and larger leg muscles indicate more rapid turns in tyrannosaurids than in other large theropods. PeerJ *7*, e6432. https://doi.org/10.7717/peerj.6432.
52. DePalma, R.A., II, Burnham, D.A., Martin, L.D., Rothschild, B.M., and Larson, P.L. (2013). Physical evidence of predatory behavior in *Tyrannosaurus rex*. Proc. Natl. Acad. Sci. USA *110*, 12560–12564. https://doi.org/10.1073/pnas.1216534110.
53. Foster, W., Brusatte, S.L., Carr, T.D., Williamson, T.E., Yi, L., and Lü, J. (2021). The cranial anatomy of the long-snouted tyrannosaurid dinosaur Qianzhousaurus sinensis from the Upper Cretaceous of China. J. Vertebr. Paleontol. *41*, e1999251. https://doi.org/10.1080/02724634.2021.1999251.
54. Rowe, A.J., and Rayfield, E.J. (2024). Morphological evolution and functional consequences of giantism in tyrannosauroid dinosaurs. iScience *27*, 110679. https://doi.org/10.1016/j.isci.2024.110679.
55. Lautenschlager, S. (2015). Estimating cranial musculoskeletal constraints in theropod dinosaurs. R. Soc. Open Sci. *2*, 150495. https://doi.org/10.1098/rsos.150495.
56. Snively, E., Cotton, J.R., Ridgely, R., and Witmer, L.M. (2013). Multibody dynamics model of head and neck function in *Allosaurus* (Dinosauria, Theropoda). Palaeontol. Electron. *16*, 1–29. https://doi.org/10.26879/338.
57. Schroeder, K., Lyons, S.K., and Smith, F.A. (2021). The influence of juvenile dinosaurs on community structure and diversity. Science *371*, 941–944. https://doi.org/10.1126/science.abd9220.
58. Myhrvold, N., Sereno, P.C., Baumgart, S.L., Formoso, K.K., Vidal, D., Fish, F.E., and Henderson, D.M. (2022). Spinosaurids as 'subaqueous foragers' undermined by selective sampling and problematic statistical inference. Preprint at bioRxiv. https://doi.org/10.1101/2022.04.13.487781.
59. Myhrvold, N.P., Baumgart, S.L., Vidal, D., Fish, F.E., Henderson, D.M., Saitta, E.T., and Sereno, P.C. (2024). Diving dinosaurs? Caveats on the use of bone compactness and pFDA for inferring lifestyle. PLoS One *19*, e0298957. https://doi.org/10.1371/journal.pone.0298957.
60. Hone, D.W.E., and Holtz, T.R., Jr. (2021). Evaluating the ecology of *Spinosaurus*: Shoreline generalist or aquatic pursuit specialist? Palaeontol. Electron. *24*, 1–28. https://doi.org/10.26879/1110.
61. Fabbri, M., Navalón, G., Benson, R.B.J., Pol, D., O'Connor, J., Bhullar, B.-A.S., Erickson, G.M., Norell, M.A., Orkney, A., Lamanna, M.C., et al.

CellPress
OPEN ACCESS

(2022). Sinking a giant: quantitative macroevolutionary comparative methods debunk qualitative assumptions. Preprint at bioRxiv. https://doi.org/10.1101/2022.05.05.490811.

62. Sereno, P.C., Myhrvold, N., Henderson, D.M., Fish, F.E., Vidal, D., Baumgart, S.L., Keillor, T.M., Formoso, K.K., and Conroy, L.L. (2022). *Spinosaurus* is not an aquatic dinosaur. eLife *11*, e80092. https://doi.org/10.7554/eLife.80092.
63. Cuff, A.R., and Rayfield, E.J. (2013). Feeding mechanics in spinosaurid theropods and extant crocodilians. PLoS One *8*, e65295. https://doi.org/10.1371/journal.pone.0065295.
64. Buffetaut, E. (1989). New remains of the enigmatic dinosaur *Spinosaurus* from the Cretaceous of Morocco and the affinities between *Spinosaurus* and *Baryonyx*. N. Jahrb. Geol. Paläontol. Monatsh. *1989*, 79–87. https://doi.org/10.1127/njgpm/1989/1989/79.
65. Charig, A.J., and Milner, A.C. (1997). *Baryonyx walkeri*, a fish-eating dinosaur from the Wealden of Surrey. Bull. Nat. Hist. Museum Lond. *53*, 11–70.
66. D'Amore, D.C., Johnson-Ransom, E., Snively, E., and Hone, D.W.E. (2025). Prey size and ecological separation in spinosaurid theropods based on heterodonty and rostrum shape. Anat. Rec. (Hoboken) *308*, 1331–1348. https://doi.org/10.1002/ar.25563.
67. Brusatte, S.L., Sakamoto, M., Montanari, S., and Harcourt Smith, W.E.H. (2012). The evolution of cranial form and function in theropod dinosaurs: insights from geometric morphometrics. J. Evol. Biol. *25*, 365–377. https://doi.org/10.1111/j.1420-9101.2011.02427.x.
68. Lautenschlager, S. (2022). Functional and ecomorphological evolution of orbit shape in Mesozoic archosaurs is driven by body size and diet. Commun. Biol. *5*, 754. https://doi.org/10.1038/s42003-022-03706-0.
69. Witmer, L.M. (2001). Nostril position in dinosaurs and other vertebrates and its significance for nasal function. Science *293*, 850–853. https://doi.org/10.1126/science.1062681.
70. Rauhut, O.W.M., and Pol, D. (2019). Probable basal allosauroid from the early Middle Jurassic Cañadón Asfalto Formation of Argentina highlights phylogenetic uncertainty in tetanuran theropod dinosaurs. Sci. Rep. *9*, 18826. https://doi.org/10.1038/s41598-019-53672-7.
71. Benson, R.B.J. (2010). A description of *Megalosaurus bucklandii* (Dinosauria: Theropoda) from the Bathonian of the UK and the relationships of Middle Jurassic theropods. Zool. J. Linn. Soc. *158*, 882–935. https://doi.org/10.1111/j.1096-3642.2009.00569.x.
72. Carrano, M.T., Benson, R.B.J., and Sampson, S.D. (2012). The phylogeny of Tetanurae (Dinosauria: Theropoda). J. Syst. Palaeontol. *10*, 211–300. https://doi.org/10.1080/14772019.2011.630927.
73. Sereno, P.C., Beck, A.L., Dutheil, D.B., Gado, B., Larsson, H.C.E., Lyon, G.H., Marcot, J.D., Rauhut, O.W.M., Sadleir, R.W., Sidor, C.A., et al. (1998). A long-snouted predatory dinosaur from Africa and the evolution of spinosaurids. Science *282*, 1298–1302. https://doi.org/10.1126/science.282.5392.1298.
74. Smith, J.B., Lamanna, M.C., Mayr, H., and Lacovara, K.J. (2006). New information regarding the holotype of *Spinosaurus aegyptiacus*. BioOne *80*, 400–406.
75. Soto, M., Toriño, P., and Perea, D. (2020). A large sized megalosaurid (Theropoda, Tetanurae) from the late Jurassic of Uruguay and Tanzania. J. S. Am. Earth Sci. *98*, 102458. https://doi.org/10.1016/j.jsames.2019.102458.
76. Paul, G.S. (2010). The Princeton Field Guide to Dinosaurs (Princeton University Press), pp. 93–94.
77. Bates, K.T., Falkingham, P.L., Breithaupt, B.H., Hodgetts, D., Sellers, W.I., and Manning, P.L. (2009). How big was 'Big Al'? Quantifying the effect of soft tissue and osteological unknowns on mass predictions for *Allosaurus* (Dinosauria: Theropoda). Palaeontol. Electron. *12*, 33p.
78. Hendrickx, C., and Mateus, O. (2014). *Torvosaurus gurneyi* n. sp., the largest terrestrial predator from Europe, and a proposed terminology of the maxilla anatomy in nonavian theropods. PLoS One *9*, e88905. https://doi.org/10.1371/journal.pone.0088905.
79. Seebacher, F. (2001). A new method to calculate allometric length-mass relationships of dinosaurs. J. Vertebr. Paleontol. *21*, 51–60. https://doi.org/10.1671/0272-4634(2001)021[0051:ANMTCA]2.0.CO;2.
80. Sereno, P.C., Tan, L., Brusatte, S.L., Kriegstein, H.J., Zhao, X., and Cloward, K. (2009). Tyrannosaurid skeletal design first evolved at small body size. Science *326*, 418–422. https://doi.org/10.1126/science.1177428.
81. Molina-Pérez, R., Larramendi, A., Connolly, D., Ramírez Cruz, G.Á., Mazzei, S., and Atuchin, A. (2019). Dinosaur Facts and Figures: The Theropods and Other Dinosauriformes (Princeton University Press). https://doi.org/10.1515/9780691190594.
82. Rayfield, E.J., Norman, D.B., Horner, C.C., Horner, J.R., Smith, P.M., Thomason, J.J., and Upchurch, P. (2001). Cranial design and function in a large theropod dinosaur. Nature *409*, 1033–1037. https://doi.org/10.1038/35059070.
83. Campione, N.E., Evans, D.C., Brown, C.M., and Carrano, M.T. (2014). Body mass estimation in non-avian bipeds using a theoretical conversion to quadruped stylopodial proportions. Methods Ecol. Evol. *5*, 913–923. https://doi.org/10.1111/2041-210X.12226.
84. Therrien, F., Henderson, D.M., and Ruff, C. (2005). Bite me – Biomechanical models of theropod mandibles and implications for feeding behavior. 228. In The Carnivorous Dinosaurs, K. Carpenter, ed. (Indiana University Press), pp. 179–198.
85. Hutchinson, J.R., Bates, K.T., Molnar, J., Allen, V., and Makovicky, P.J. (2011). A computational analysis of limb and body dimensions in *Tyrannosaurus rex* with implications for locomotion, ontogeny, and growth. PLoS One *6*, e26037. https://doi.org/10.1371/journal.pone.0026037.
86. Sues, H.D., Frey, E., Martill, D.M., and Scott, D.M. (2002). *Irritator challengeri*, a spinosaurid (Dinosauria: Theropoda) from the Lower Cretaceous of Brazil. J. Vertebr. Paleontol. *22*, 535–547. https://doi.org/10.1671/0272-4634(2002)022[0535:ICASDT]2.0.CO;2.
87. Zapata, U., Metzger, K., Wang, Q., Elsey, R.M., Ross, C.F., and Dechow, P.C. (2010). Material properties of mandibular cortical bone in the American alligator, *Alligator mississippiensis*. Bone *46*, 860–867. https://doi.org/10.1016/j.bone.2009.11.010.
88. Porro, L.B., Holliday, C.M., Anapol, F., Ontiveros, L.C., Ontiveros, L.T., and Ross, C.F. (2011). Free body analysis, beam mechanics, and finite element modeling of the mandible of *Alligator mississippiensis*. J. Morphol. *272*, 910–937. https://doi.org/10.1002/jmor.10957.
89. Ballell, A., Moon, B.C., Porro, L.B., Benton, M.J., and Rayfield, E.J. (2019). Convergence and functional evolution of longirostry in crocodylomorphs. Palaeontology *62*, 867–887. https://doi.org/10.1111/pala.12432.
90. Herbst, E.C., Lautenschlager, S., Bastiaans, D., Miedema, F., and Scheyer, T.M. (2021). Modeling tooth enamel in FEA comparisons of skulls: Comparing common simplifications with biologically realistic models. iScience *24*, 103182. https://doi.org/10.1016/j.isci.2021.103182.
91. Holliday, C.M. (2009). New insights into dinosaur jaw muscle anatomy. Anat. Rec. (Hoboken) *292*, 1246–1265. https://doi.org/10.1002/ar.20982.
92. Sakamoto, M. (2006). Scaling bite force in predatory animals: How does *T. rex* compare with living predators? J. Vertebr. Paleontol. *26*, 118A.
93. R Core Team (2021). R: A Language and Environment for Statistical Computing (R Foundation for Statistical Computing).
94. Marcé-Nogué, J., De Esteban-Trivigno, S., Escrig, C., and Gil, L. (2016). Accounting for differences in element size and homogeneity when comparing Finite Element models: Armadillos as a case study. Palaeontol. Electron. *19*, 1–22. https://doi.org/10.26879/609.
95. Morales-García, N.M., Burgess, T.D., Hill, J.J., Gill, P.G., and Rayfield, E.J. (2019). The use of extruded finite-element models as a novel alternative to tomography-based models: a case study using early mammal jaws. J. R. Soc. Interface *16*, 20190674. https://doi.org/10.1098/rsif.2019.0674.
96. Ballell, A., and Ferrón, H.G. (2021). Biomechanical insights into the dentition of megatooth sharks (Lamniformes: Otodontidae). Sci. Rep. *11*, 1232. https://doi.org/10.1038/s41598-020-80323-z.
97. Paradis, E., and Schliep, K. (2019). ape 5.0: An environment for modern phylogenetics and evolutionary analyses in R. Bioinformatics *35*, 526–528. https://doi.org/10.1093/bioinformatics/bty633.

STAR★METHODS

KEY RESOURCES TABLE

REAGENT or RESOURCE	SOURCE	IDENTIFIER
Software and algorithms		
Abaqus/CAE	https://www.3ds.com/products/simulia/abaqus/cae/	Abaqus/CAE Version 6.14-1
Altair HyperMesh	https://altair.com/	HyperMesh Version 2021
Artec Studio Professional	https://www.artec3d.com/3d-software/artec-studio/	Artec Studio Professional Version 14
Avizo Lite	https://www.thermofisher.com/uk/	Avizo Lite Version 9.5
Blender	https://www.blender.org/	Blender Version 2.82
Geomagic Studio	https://www.3dsystems.com/software/	Geomagic Studio Version 12
MeshLab	https://www.meshlab.net/	MeshLab 2020.06
RStudio	RStudio Team https://www.rstudio.com/	RStudio Version 2023.06.1

EXPERIMENTAL MODEL AND STUDY PARTICIPANT DETAILS

Taxonomic Sampling

Megalosauroidea

Megalosauroidea (also referred to as Spinosauroidea) is a clade of tetanuran theropods that lived from the Middle Jurassic to the Late Cretaceous and may be paraphyletic with respect to Allosauroidea.[70] They represent one of the first major radiations of giant theropod dinosaurs.[71] The possibly semi-aquatic *Spinosaurus* may have been the longest bipedal dinosaur, with some estimates exceeding 14 m.[62] There is evidence for body size increase through time within the clade: basal taxa from the Early Jurassic were smaller than later Middle Jurassic forms.[72] The largest members—*Spinosaurus* and *Suchomimus*—are from North Africa,[73,74] but megalosauroids are also known from North America, South America, and possibly Asia.[75] The taxa included in this study were *Torvosaurus*, *Spinosaurus*, and *Suchomimus* (Table 1).

Allosauroidea

Allosauroids are tetanuran theropods whose earliest known representative is *Shidaisaurus* from Aalenian-aged deposits (~174 Ma) in Middle Jurassic China.[39] Among the latest-surviving taxa is *Carcharodontosaurus* from the Cenomanian of North Africa (~100 Ma), which may have coexisted and competed with *Spinosaurus*. Another large allosauroid, *Giganotosaurus*, lived in Argentina during the same interval and likely served as a generalized predator.[84] Allosauroids had a wide geographic range across both hemispheres during the Jurassic and Early Cretaceous but were later displaced by tyrannosaurids in the Late Cretaceous.[1] Distinctive features include relatively long forelimbs, large orbits, and narrow skulls. Their teeth and skull morphology suggest a slicing feeding style, similar to Komodo dragons,[82] contrasting with the bone-crushing bites of tyrannosaurids, which are more akin to those of extant crocodilians.[49] This study included *Monolophosaurus*, *Allosaurus*, *Neovenator*, *Giganotosaurus*, and *Acrocanthosaurus* (Figure 1 and Table 1).

Tyrannosauroidea

Tyrannosauroids are coelurosaurian theropods that first appeared in the Oxfordian of the Middle Jurassic (~160 Ma) in Asia and later dispersed to North America.[45] Large, derived tyrannosaurids such as *Tyrannosaurus* exhibit broad skulls, reduced forelimbs, and long legs relative to body size. Mass estimates for *Tyrannosaurus* are the highest for any known theropod, ranging from 6,000 to 9,500 kg.[8,85] Some lineages, such as the alioramins, retained gracile morphologies into adulthood—potentially reflecting ecological differentiation and separation of resource use in Late Cretaceous ecosystems. Such divergence may have reduced interspecific competition and enabled the coexistence of multiple apex predators within a single faunal assemblage.[15] Owing to their excellent fossil record and public interest, tyrannosauroids are among the most studied theropods, particularly in terms of feeding biomechanics.[29,32,41,48,49,54] Nevertheless, few studies have compared these biomechanical traits in a broad macroevolutionary context. The taxa sampled here were *Raptorex, Bistahieversor, Albertosaurus, Alioramus, Daspletosaurus*, and two specimens of *Tyrannosaurus* (Figure 1 and Table 1).

METHOD DETAILS

3D imaging and model generation

Skull lengths and 3D models were compiled from the literature and specimen scanning to assess potential relationships between size and cranial stress (Tables 1 and S1). Models were generated using either CT scanning or structured-light surface scanning[35] (Table S2), depending on the specimen's size and availability. Surface scanning was preferred for mounted skeletons or large specimens, which are often inaccessible for CT scanning.

Models were derived from original fossils and high-quality casts. Some were composites due to missing anatomical elements, which were reconstructed using closely related taxa. For instance, missing cranial bones in the *Suchomimus* model were based on the Brazilian spinosaurid *Irritator*,[86] and missing elements in *Torvosaurus* were modeled after related megalosauroids.

Scanning was performed at multiple institutions: *Allosaurus* was CT scanned from original material; *Spinosaurus* and *Giganotosaurus* were CT scanned from casts. Surface scans were performed at the Dinosaur Discovery Museum (DDM) using elevated platforms for mounted specimens (*Suchomimus*, *Torvosaurus*, *Acrocanthosaurus*, *Ceratosaurus*, *Herrerasaurus*), at the Milwaukee Public Museum for *Monolophosaurus*, and at the Science Museum of Minnesota for *Dilophosaurus*. The *Neovenator* model was based on a museum sculpture, with missing regions informed by *Allosaurus* morphology (M. Munt, pers. comm., 2021). Despite varying model sources, interspecific differences were expected to outweigh any cast-specific discrepancies.

Model processing and editing

Surface scans were processed in Artec Studio 14 Professional, using real-time fusion at 7–8 frames/sec. Crania and mandibles were scanned separately, aligned manually, and merged into single STL files. Frames with high error, stray pixels, and non-cranial elements (e.g., cervical vertebrae) were removed. Global Registration and Sharp Fusion were applied to preserve detail and create watertight, polygonal models. Floating pixels and holes were corrected using in-built filters.

The *Dilophosaurus* model, based on a half-skull resin cast, was mirrored and reconstructed in Blender 2.82, then cleaned of intersecting triangles in Geomagic Studio 12. Final edits, including hole filling and mesh correction, were also completed in Geomagic Studio 12. At this stage, model surface area, triangle count, element count, and volume were measured and standardized across specimens to reduce potential analytical bias (Tables S1 and S2).

Several specimens posed reconstruction challenges. *Acrocanthosaurus* was part of a full skeletal mount on public display at the DDM. The skull was centrally positioned and elevated, necessitating ladder access and scanning at awkward angles with limited maneuverability. As a result, aspects of the cranial surface exhibit areas of low-resolution and accuracy due to restricted scanner coverage, which appears in the finalized 3D model. The skull roof of *Daspletosaurus* was similarly difficult to interpret due to complex surface topography and elevation. In *Bistahieversor*, fine details of the parietal region were reconstructed by referencing *Tyrannosaurus rex* (FMNH PR 2081).

The internal anatomy of *Torvosaurus* and *Suchomimus* was reconstructed using anatomical comparisons with *Spinosaurus* due to a lack of preserved internal cranial features. While general morphology was retained, the endocranial surfaces and internal structures may be simplified or exhibit ‘blocky’ geometries.

Several specimens, including *Acrocanthosaurus*, *Suchomimus*, *Torvosaurus*, *Herrerasaurus*, *Ceratosaurus*, and *Monolophosaurus* were scanned while articulated with cervical vertebrae as part of full skeletal mounts. To isolate the cranium, the anterior cervical vertebrae (C1–C2 in nearly all models) were digitally removed post hoc, and the occipital region—including the occipital condyle, basioccipital, and posterior portions of the parietals and squamosals—was reconstructed based on preserved morphology and comparisons with related taxa.

The *Alioramus* skull was disarticulated and previously CT scanned as approximately 30 individual elements, each provided digitally as separate files. These were imported and aligned in Blender 2.82 through careful manual articulation, with anatomical orientation guided by osteological landmarks and comparison with related tyrannosaurids.[54]

Finite element analysis (FEA)

Meshing was conducted in HyperMesh (Altair), using tetrahedral elements with 10 mm size and optimized quality settings. Each model was assigned material properties based on crocodilian bone (Young’s modulus = 15,000 MPa; Poisson’s ratio = 0.29) to approximate dinosaur bone.[87–89] Dentine properties were not modeled, as they contribute minimally to stress distribution in comparative analyses.[90]

Constraint nodes were placed at the quadrate (crania) and articular (mandibles) to prevent rigid body motion, and additional constraints were applied to the premaxillary teeth to simulate prey contact.[91] Muscle forces were estimated based on attachment sites from the literature[32,54,61] (Figures S3–S5); forces were scaled from *Tyrannosaurus* (FMNH PR 2081) using the subtemporal fenestra method.[92] Muscle cross-sectional area was measured in ImageJ and multiplied by an isometric muscle stress of 31.5 N/cm^2 (Figure S6 and Table S3).

To normalize body size and isolate the effects of skull shape, we scaled each model’s muscle force relative to its surface area (Table S4). FEA simulations were rerun at both actual and scaled sizes, and von Mises stress and maximum principal strain were visualized using 3D stress maps with a 0–25 MPa stress range. Skull and mandible stress values were analyzed separately, given the fragility of mandibles.

Limitations of the Study

One limitation of this study pertains to the anatomical reconstructions of surface scanned skulls. As this scanning method only captures the external geometries of objects, we must use figures from the literature and existing CT data to reconstruct the internal anatomy of surface scanned-object files. CT-derived 3D models are likely more reflective of biological reality due to the preservation of air-filled spaces in the diapsid skull, though surface scanning was previously tested and found to be reliable in comparative studies in Rowe and Rayfield,[35] particularly when assessing overall patterns of stress and strain. Additionally, several cranial models were affected by scanning constraints or limited fossil preservation, which may introduce minor inaccuracies. For example, the

Acrocanthosaurus model was derived from a mounted specimen scanned under difficult conditions, limiting surface fidelity in some regions.

While the finite element results are broadly consistent across lineages, some aspects of stress magnitude and distribution are influenced by reconstruction choices, including muscle origin and insertion sites. We based our muscle placements on previous reconstructions[32,54] to produce conservative and repeatable estimates. However, alternative reconstructions exist; for example, those recently proposed by Johnson-Ransom et al.[41] which reflect different interpretations of soft tissue anatomy. Although such differences are unlikely to overturn our main conclusions, future comparative FEA studies would be valuable to test how varying muscle topologies affect cranial stress patterns.

Data analysis

Mesh-weighted arithmetic mean (MWAM) von Mises stress values were calculated in RStudio[93] to account for mesh resolution and element volume.[94–96] Models with extensive editing, e.g., *Dilophosaurus*, were interpreted cautiously, as prior work suggests such models may yield elevated stress values.[35] Final data were plotted against skull length to assess the relationship between size and stress (Figures 3, 5, S1, and S2).

QUANTIFICATION AND STATISTICAL ANALYSIS

Statistical significance was tested using p-values and phylogenetic generalized least squares (PGLS) regression in RStudio with the 'ape' package.[97] Regression outputs are presented in the Results section; N=18 and represents the total number of dinosaur taxa. No additional statistical tests were applied.[76–81,83]

ADDITIONAL RESOURCES

No additional resources were used in this study.

Current Biology, Volume *35*

Supplemental Information

Carnivorous dinosaur lineages adopt different skull performances at gigantic size

Andre J. Rowe and Emily J. Rayfield

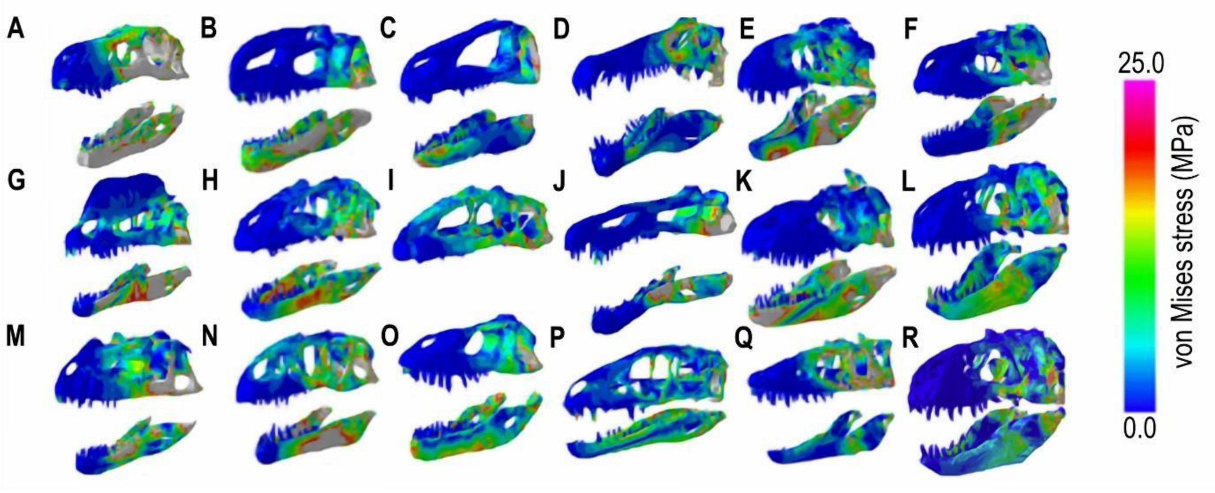

Figure S1. Larger FE models of the actual size results. Related to Figure 2.

(A) *Herrerasaurus*, (B) *Monolophosaurus*, (C) *Acrocanthosaurus*, (D) *Spinosaurus*, (E) *Bistahieversor*, (F) *Daspletosaurus*, (G) *Dilophosaurus*, (H) *Allosaurus,* (I) *Giganotosaurus,* (J) *Suchomimus,* (K) *Albertosaurus,* (L) *Tyrannosaurus* (USNM 555000), (M) *Ceratosaurus*, (N) *Neovenator*, (O) *Torvosaurus,* (P) *Raptorex*, (Q) *Alioramus*, (R) *Tyrannosaurus* (FMNH PR 2081).

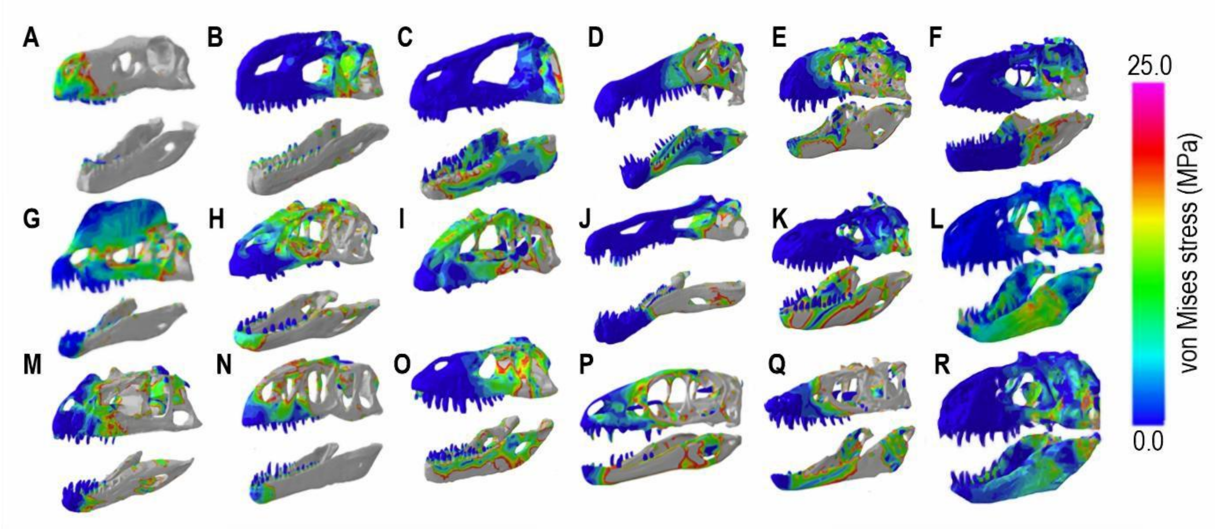

Figure S2. Larger FE models of the size corrected results. Related to Figure 4.

(A) *Herrerasaurus*, (B) *Monolophosaurus*, (C) *Acrocanthosaurus*, (D) *Spinosaurus*, (E) *Bistahieversor*, (F) *Daspletosaurus*, (G) *Dilophosaurus*, (H) *Allosaurus,* (I)

Giganotosaurus, (J) *Suchomimus,* (K) *Albertosaurus,* (L) *Tyrannosaurus* (USNM 555000), (M) *Ceratosaurus*, (N) *Neovenator*, (O) *Torvosaurus,* (P) *Raptorex*, (Q) *Alioramus*, (R) *Tyrannosaurus* (FMNH PR 2081).

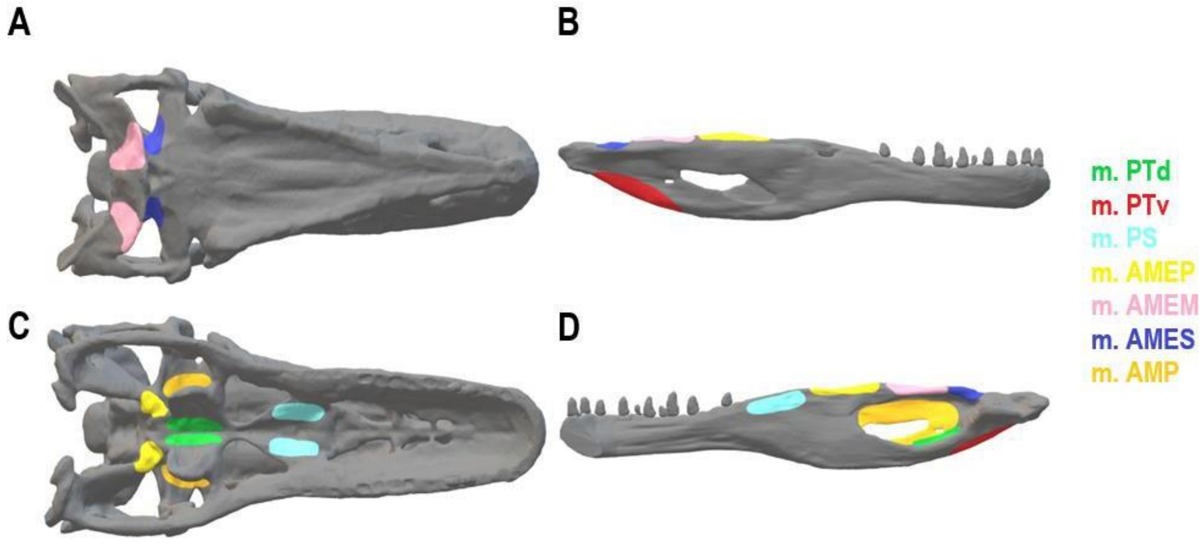

Figure S3. Locations of muscle insertions for *Allosaurus jimmadseni* (MOR 693) to represent muscle mapping for allosauroids in this study. Related to STAR*Methods.

(A) Cranium in dorsal view, (B) mandible in lateral view, (C) cranium in ventral view, and (D) mandible in medial view. Muscle abbreviations: m. PTd, M. pterygoideus dorsalis; m. PTv, M. pterygoideus ventralis; m. PS, M. pseudotemporalis complex; m. AMEP, M. adductor mandibulae externus profundus; m. AMEM, Musculus adductor mandibulae externus medialis; m. AMES, M. adductor mandibulae externus superficialis; m. AMP, M. adductor mandibulae posterior.

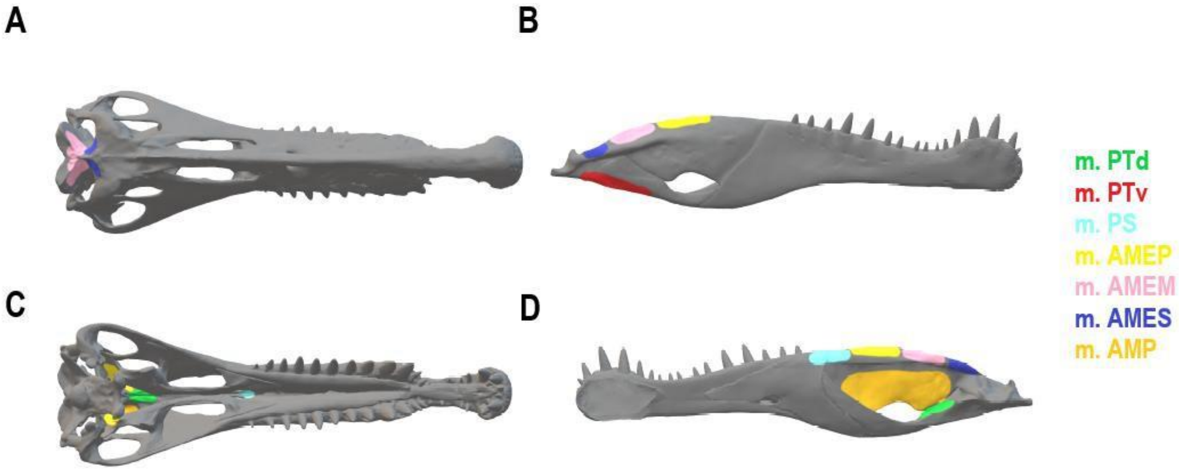

Figure S4. Locations of muscle insertions for *Spinosaurus aegyptiacus* (FSAC-KK 11888) to represent muscle mapping for megalosauroids in this study. Related to STAR*Methods.

(A) Cranium in dorsal view, (B) mandible in lateral view, (C) cranium in ventral view, and (D) mandible in medial view.

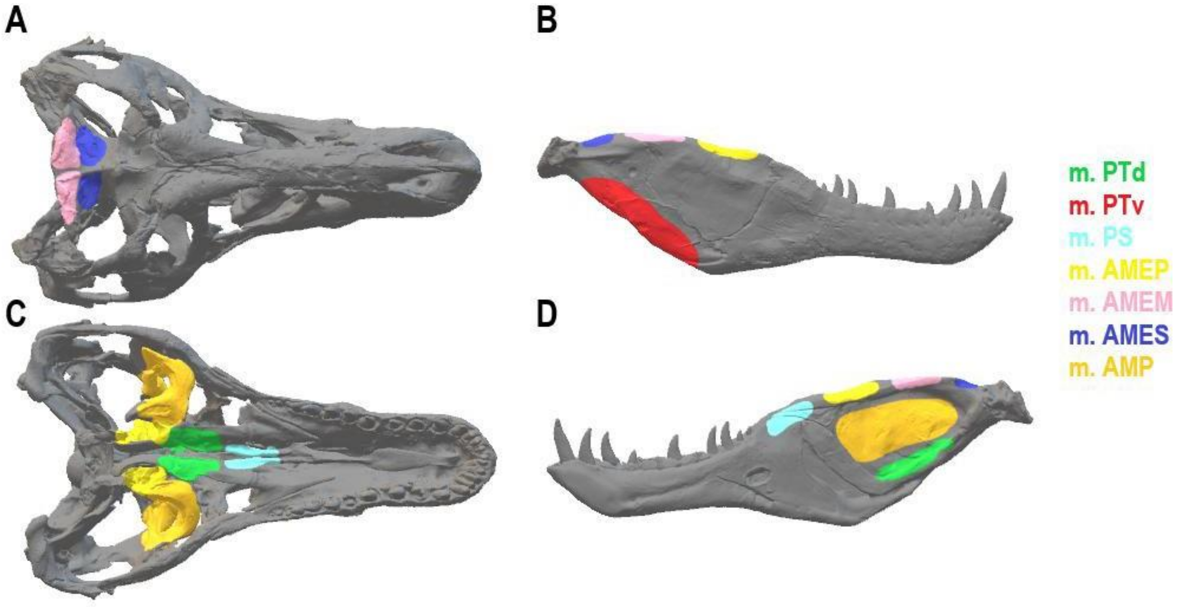

Figure S5. Locations of muscle insertions for *Tyrannosaurus rex* (FMNH PR 2081) to represent muscle mapping for tyrannosauroids in this study. Related to STAR*Methods.

(A) Cranium in dorsal view, (B) mandible in lateral view, (C) cranium in ventral view, and (D) mandible in medial view.

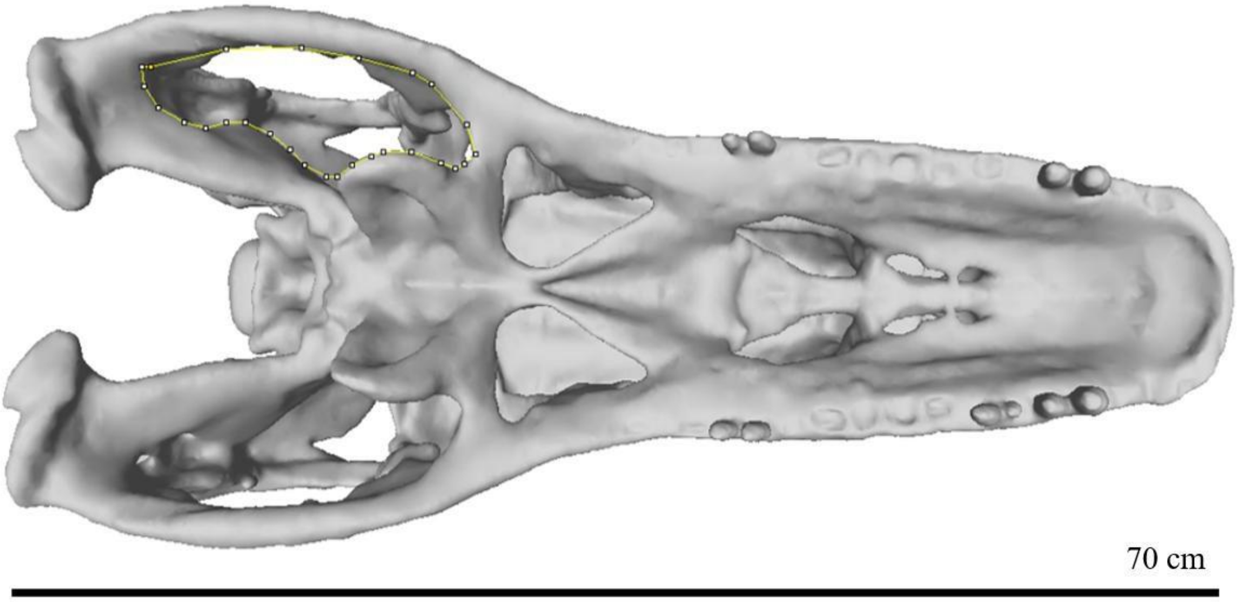

Figure S6. ***Allosaurus jimmadseni*** **(MOR 693) cranium in ventral view, with one adductor chamber measured in ImageJ (Fiji). Related to STAR*Methods.**

Adductor chamber measurements were multiplied by isometric muscle tension 31.5 N/cm^2 to calculate muscle force components applied in Abaqus/CAE 6.14-1.

Specimen	Cranial triangle count	Mandibular triangle count	Cranial element count	Mandibular element count	Cranial volume (mm³)	Mandibular volume (mm³)
Herrerasaurus ischigualastensis	350,006	315,016	525,009	472,524	174,985	157,506
Dilophosaurus wetherilli	349,972	299,972	524,958	449,958	174,974	149,984
Ceratosaurus nasicornis	398,798	314,918	598,197	472,377	199,391	157,459
Monolophosaurus jiangi	369,856	309,978	554,784	464,967	184,912	154,991
Allosaurus jimmadseni	155,102	114,838	232,613	172,257	77,493	57,411
Neovenator salerii	599,992	499,990	899,988	749,985	299,974	249,997
Acrocanthosaurus atokensis	400,014	350,026	600,021	525,039	200,001	175,015
Giganotosaurus carolinii	97,534	N/A	146,301	N/A	248,741	N/A
Torvosaurus gurneyi	499,234	400,066	748,851	600,099	249,613	200,031

Spinosaurus aegyptiacus	366,500	229,448	549,750	344,172	285,040	214,716
Suchomimus tenerensis	499,980	400,008	749,970	600,012	249,988	200,000
Raptorex kriegsteini	289,478	204,770	434,217	307,155	144,711	102,385
Bistahieversor sealeyi	923,682	529,002	1,385,523	793,503	461,821	264,485
Albertosaurus sarcophagus	651,876	317,422	977,814	476,133	325,886	158,705
Alioramus altai	465,994	38,102	698,991	57,153	227,012	19,045
Daspletosaurus torosus	386,920	467,596	580,380	956,394	616,518	318,802
Tyrannosaurus rex (USNM 555000)	1,270,954	711,910	1,906,431	1,067,865	635,435	355,957
Tyrannosaurus rex (FMNH PR 2081)	1,284,114	860,746	1,926,171	1,291,119	642,017	430,375

Table S1. 3D model triangles, elements, and volume measurements (mm^3). Related to STAR*Methods.

All values were calculated in MeshLab 2020.06.

Specimen name	Cranial model surface area (mm^2)	Mandible model surface area (mm^2)	Replica of skull?
Herrerasaurus ischigualastensis	94382	51683	Yes
Dilophosaurus wetherilli	608045	184758	Yes
Ceratosaurus nasicornis	360584	182932	Yes
Monolophosaurus jiangi	551013	249787	Yes
Allosaurus jimmadseni	788710	306512	Yes
Neovenator salerii	567398	245641	No
Acrocanthosaurus atokensis	1545905	1169611	Yes
Giganotosaurus carolinii	1880562	N/A	Yes
Torvosaurus gurneyi	1815301	869156	Yes
Spinosaurus aegyptiacus	2257382	1575902	Yes
Suchomimus tenerensis	828898	677021	Yes

Raptorex kriegsteini	904346	49811	Yes
Bistahieversor sealeyi	1777871	885024	No
Albertosaurus sarcophagus	1382871	677021	Yes
Alioramus altai	457843	160486	No
Daspletosaurus torosus	1526939	803412	Yes
Tyrannosaurus rex (USNM 555000)	4600059	1921720	Yes
Tyrannosaurus rex (FMNH PR 2081)	4604695	1924344	No

Table S2. Surface area values (mm^2) for the cranial and mandibular models of each taxon and the replica status of each skull included. Related to STAR*Methods.

All values were calculated in MeshLab 2020.06.
